# Systematic Review and Meta-Analysis on Randomized Controlled Trials on Efficacy and Safety of Panax Notoginseng Saponins in Treatment of Acute Ischemic Stroke

**DOI:** 10.1155/2021/4694076

**Published:** 2021-07-09

**Authors:** Liu-ding Wang, Zhen-min Xu, Xiao Liang, Wen-ran Qiu, Shao-jiao Liu, Ling-ling Dai, Ye-fei Wang, Chun-yan Guo, Xiang-hua Qi, Jian Wang, Yan-bing Ding, Yun-ling Zhang, Xing Liao

**Affiliations:** ^1^Xiyuan Hospital, China Academy of Chinese Medical Sciences, Beijing 100091, China; ^2^Center for Evidence-based Chinese Medicine, Institute of Basic Research in Clinical Medicine, China Academy of Chinese Medical Sciences, Beijing 100700, China; ^3^Graduate School, Beijing University of Chinese Medicine, Beijing 100029, China; ^4^The Affiliated Hospital of Shandong University of Traditional Chinese Medicine, Jinan 250014, China; ^5^The Affiliated Hospital to Changchun University of Chinese Medicine, Changchun 130021, China; ^6^Chinese Medicine Hospital of Hubei Province, Wuhan 430074, Hubei, China

## Abstract

**Objective:**

To assess the efficacy and safety of PNS on antiplatelet therapy in the treatment of AIS.

**Methods:**

We searched 7 literature databases and 2 clinical studies databases for randomized controlled studies (RCTs) evaluating PNS as an adjuvant therapy for AIS. Relevant studies were retrieved and screened, and data were extracted independently by two reviewers. The quality of the included studies was assessed using the Cochrane Risk Assessment Tool. Meta-analysis was carried out with the Rev Man 5.4 software.

**Results:**

Of 8267 records identified, 43 RCTs met our inclusion criteria (*n* = 4170 patients). Patients assigned to PNS with conventional treatments (CTs) had improved functional independence at 90 days compared with those assigned to CTs alone (*RR* = 1.87, 95% *CI* = 1.37, to 2.55, *P* < 0.0001). Patients who received PNS combined with CTs showed significantly high improvements in neurological function among individuals with AIS on the neurologic deficit score (NDS) (*MD*_*CSS*_ = −5.71, 95% *CI* = −9.55 to −1.87, *P*=0.004; *MD*_*NIHSS*_ = −3.94, 95% *CI* = −5.65 to −2.23, *P* < 0.00001). The results also showed PNS contributed to a betterment in activities of daily living (ADL) on the Barthel index (*MD*_*day*__10 *BI*_ = 4.86, 95% *CI* = 2.18, to 7.54, *P* < 0.00001; *MD*_*day 14 BI*_ = 13.92, 95% *CI* = 11.46 to 16.38, *P* < 0.00001; *MD*_*day 28 BI*_ = 7.16, 95% *CI* = 0.60, to 13.72, *P* < 0.00001). In addition, PNS, compared with CTs alone, could significantly improve overall response rate (ORR) (*RR*_*NIHSS*_ = 1.20, 95% *CI* = 1.16, to 1.24, *P* < 0.00001; *RR*_*CSS*_ = 1.15, 95% *CI* = 1.08, to 1.24, *P* < 0.0001), hemorheological parameters, maximum platelet aggregation rate (MPAR) (*MD* = −6.82, 95% *CI* = −9.62 to −4.02, *P* < 0.00001), platelet parameters (*MD*_*PLT*_ = 4.85, 95% *CI* = 1.82 to 7.84, *P*=0.002; *MD*_*MPV*_ = −0.79, 95% *CI* = −1.09 to −0.48, *P* < 0.00001), and serum CD62P (*MD* = −0.21, 95% *CI* = −0.29 to −0.13, *P* < 0.00001). The incidence of adverse reactions in PNS was lower than that in the control group (*RR* = 0.62, 95% *CI* = 0.39 to 0.97, *P*=0.04). Adverse reactions in the PNS were mild adverse reactions.

**Conclusion:**

PNS may be effective and safe in treating AIS on ameliorating neurological deficit, improving activities of daily living function, and enhancing antiplatelet effects. However, more high-quality evidence is needed before it can be recommended for routine antiplatelet therapy in patients with AIS.

## 1. Introduction

Acute ischemic stroke (AIS), also known as acute cerebral infarction (ICD10 Code: i63.902), is a life-threatening medical condition with a high incidence that carries a grave prognosis if not addressed promptly. It is characterized by acute onset. According to epidemiological studies, there are about 17 million patients with AIS in the world every year [1], and 6.2 million people die from AIS [2]. The mortality and disability rate of patients with AIS in China is 34.5%–37.1% within 3 months after the onset of disease [3, 4]. Its pathogenesis is sudden occlusion of the cerebral artery, resulting in cerebrovascular circulation dysfunction and irreversible neuronal necrosis [5]. At present, conventional treatments recommended by clinical practice guidelines include thrombolytic drugs, antiplatelet drugs, anticoagulants, and neurotrophic drugs. However, there are side effects and drug resistance, such as intracerebral bleeding after thrombolysis [6] and clopidogrel resistance [7]. Naturally, reducing the rate of intracranial hemorrhage after reperfusion and overcoming clopidogrel resistance are the requirements of new antiplatelet drugs in the future.

Panax Notoginseng Saponins (PNS), an active ingredient extracted from Chinese herbal medicine Panax notoginseng, has been widely used in the treatment of AIS in China. Panax notoginseng is traditionally applied as an activating blood drug, also known as Sanqi or Tianqi. Sanqi was first recorded in the “Compendium of Materia Medica” (Bencao Gangmu) in 1758, in which it was called “more precious than gold” (jinbuhuan). Its preparations include Xuesaitong injection, Xueshuantong injection, Lulutong injection, Xuesaitong capsules, Xueshuantong capsules, Sanqi Tongshu capsules, and Xuesaitong dropping pills. In recent five years, systematic reviews to evaluate the efficacy of PNS have been published [8–11]. Pharmacological experiments to study the mechanism of PNS showed the effects on anti-ischemia-reperfusion injury [12]. The synergistic mechanism of Chinese herbal medicine and antiplatelet drugs of western medicine has caught worldwide attention. It was found that PNS could enhance the antiplatelet effect by regulating the arachidonic acid (AA) metabolic pathway [13], inhibiting thromboxane A2 (TXA2) [14] or aspirin hydrolase [15], and increasing the AUC_0-∞_ or Cmax of the clopidogrel active metabolite [16].

However, most of the existing systematic reviews observed a certain kind of PNS preparations [8, 11] and paid more attention to the efficacy of PNS combined with a certain western medicine [9, 10], such as Xueshuantong combined with edaravone or butylphthalide, but was lacking in the latest clinical research results to evaluate the therapeutic effect of PNS as the only variable in the intervention and control group. In this study, RCTs of PNS in the treatment of AIS were selected for systematic review and meta-analysis, in order to provide up-to-date evidence for clinical application of PNS.

## 2. Information and Methods

### 2.1. Research Registrations

This systematic review protocol was registered with PROSPERO (PROSPERO Registration: CRD42021229265). The protocol is shown in Supplementary files. 7.

### 2.2. Data Sources and Search Strategy

The current systematic review was part of the project “Identification of Priorities for Improvement of Implementation on Evidence-Based Traditional Chinese Medicine” (zz13-024-3). Based on this project, databases such as CNKI, Wanfang, VIP, CBM, EMBASE, PubMed, Cochrane Library, ClinicalTrials.gov, and ChiCTR were searched by the research team. A separate database of AIS treated with traditional Chinese medicine was established. The retrieval time is from the establishment of the database to December 2020. The database classified the literature systematically according to the types of research and intervention measures. Considering that the above search did not explicitly mention PNS, we conducted an additional search by using keywords including PNS, Xuesaitong, Xueshuantong, Sanqi Tongshu capsule, and Lulutong and supplemented the database of PNS in the treatment of AIS. Taking PubMed as an example, the specific supplementary retrieval strategies are presented in Supplementary File 1. On 4 April 2021, we updated the database search of PubMed and CNKI. We used the same search method, except that we narrowed the searches to 2020 onwards.

### 2.3. Inclusion Criteria

#### 2.3.1. Types of Studies

Randomized controlled trials (RCTs) were included.

#### 2.3.2. Types of Participants

Patients diagnosed with AIS were included.

#### 2.3.3. Types of Interventions

The experimental group treated with PNS combined with conventional treatments (CTs) and the control group treated with the same CTs were included. CTs are considered to include thrombolytic drugs, antiplatelets, anticoagulants, statins, neuroprotective agents, and antihypertensive and collateral circulation drugs.

#### 2.3.4. Types of Outcomes

Efficacy outcomes: the primary outcome was a 3-month functional independence rate (mRS scores 0–2), and the secondary outcomes were neurologic deficit score (NDS), ADL-Barthel score, overall response rate (ORR), hemorheological parameters, maximum platelet aggregation rate (MPAR), platelet parameters, CD62P, and coagulation function.

Safety outcomes: incidence of adverse reactions and adverse reactions.

### 2.4. Exclusion Criteria

We excluded trials as follows: (1) other TCM treatments were applied in either treatment or control group; (2) full texts were not available; (3) data is not complete; (4) language is not Chinese or English.

### 2.5. Study Selection

Two reviewers (LDW and ZMX) independently performed literature selection according to the predefined eligibility criteria. The records retrieved in all databases were imported into NoteExpress3.2, and the duplicated records were deleted. Records were first screened based on the title and abstract, and in cases of uncertainty, the full texts were obtained. Any disagreement between the paired reviewers was resolved through discussing with a third reviewer (XL).

### 2.6. Data Extraction

Data extraction was conducted by two reviewers (LDW and WRQ) using a standardized, predetermined data extraction form. Two reviewers independently extracted data from each trial and then cross-checked the data. Discrepancies were solved by discussion between the two reviewers or arbitrated by the senior researcher (XL) if necessary. We extracted the following data: (1) study characteristics, (2) participant's baseline characteristics and inclusion/exclusion criteria, (3) details of intervention and control groups, and (4) outcomes (dichotomous data were the number of events and total participants per group; continuous data were presented as mean, standard deviation, and total participants per group).

### 2.7. Methodological Quality Assessment

Two reviewers (LDW and SJL) independently assessed the risk of bias of the included trials. According to the Cochrane Risk of Bias tool [17], seven fields of risk of bias were evaluated as follows: random sequence generation, allocation concealment, blinding of participants and personnel, blinding of outcome assessment, incomplete outcome data, selective reporting, and other bias. The other bias includes the following aspects, comparable baseline, sample size calculation, participation of pharmaceutical enterprises, and deception. The evaluation results were ranked as “low risk,” “unclear risk,” or “high risk.” If disagreements on the assessment were identified, the researcher (XL) was consulted.

### 2.8. Data Analysis

Review Manager software (RevMan, version 5.4) was utilized to conduct the data analysis of dichotomous and continuous outcomes, which were extracted from the primary studies. Risk ratio (*RR*) was used for dichotomous data while weighted mean difference (*WMD*) or standardized mean difference (*SMD*) were adopted for continuous variables as effect size, both of which were demonstrated with effect size and 95% confidence intervals (*CI*). When no statistical heterogeneity was identified (heterogeneity test, *P* ≥ 0.10, or *I*^*2*^ ≤ 50%), a fixed-effects model was selected; otherwise, a random-effects model was applied. We would perform subgroup analyses and sensitivity analyses based on the course of treatment or dose or follow-up time. Sources of heterogeneity will be fully explored if enough data are available. A funnel plot was used to detect the publication bias if the number of included trials was larger than ten for an outcome. Statistical significance was set at *P* < 0.05.

### 2.9. Reporting Bias Assessment

To assess small-study effects, we planned to generate funnel plots for meta-analyses including at least 10 trials of varying size to detect the publication bias. To assess outcome reporting bias, we compared the outcomes specified in trial protocols with the outcomes reported in the corresponding trial publications; if trial protocols were unavailable, we compared the outcomes reported in the methods and results sections of the trial publications.

### 2.10. Certainty Assessment

Two reviewers (LDW and CYG) independently assessed the certainty of the evidence using the Grading of Recommendations Assessment, Development, and Evaluation (GRADE) approach [18] and assessed the certainty of the evidence as “high,” “moderate,” “low,” or “very low.” The certainty can be downgraded for five GRADE considerations (study limitations, consistency of effect, imprecision, indirectness, and publication bias) and upgraded for three reasons (large magnitude of an effect, dose-response gradient, and effect of plausible residual confounding).

## 3. Results

### 3.1. Study Selection

**Figure 1:**
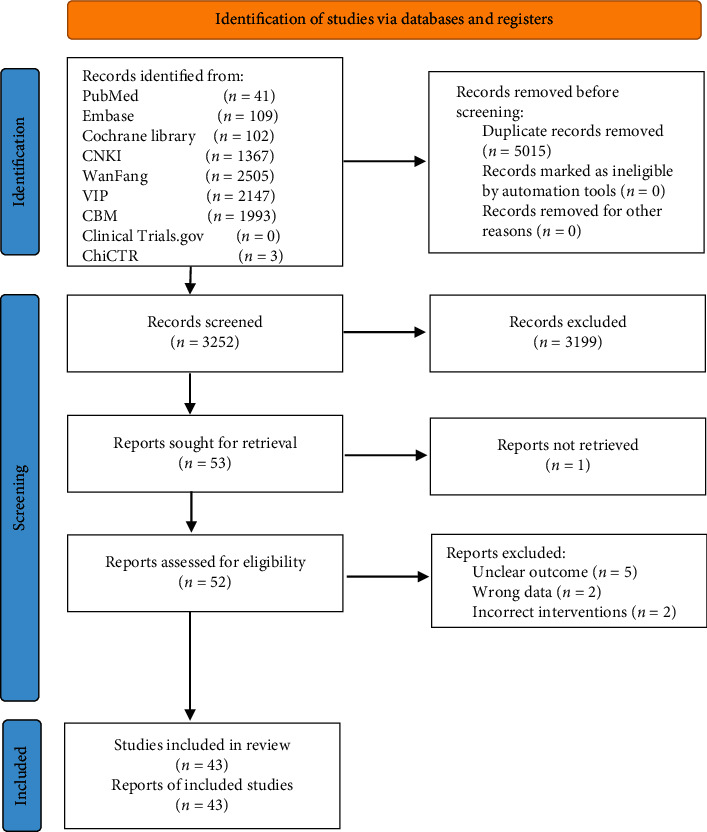
Flow diagram for identification of studies.

The search yielded 8267 records. There were 5015 duplicates, leaving 3252 to be screened by title and abstract from which 53 eligible records were retained for full-text evaluation. After careful evaluation and no disagreements between the two reviewers, 10 reports were excluded. Ultimately, 43 reports involving 4170 participants met our inclusion criteria [19–61]. See Figure 1 for details of the flow diagram with the search results and selection of studies. A list of 9 studies that might appear to meet the inclusion criteria but which were excluded, with citation and the reason for exclusion, is reported in Supplementary File 2.

### 3.2. Study Characteristics

All 43 included RCTs were performed in China. All interventions were PNS in combination with CTs. Among them, 17 studies [19, 23, 24, 28–31, 35, 36, 38–40, 44, 45, 49, 51, 61] were Xueshuantong injection, 18 [20–22, 25–27, 31, 32, 34, 37, 41–43, 46–48, 50, 52] were Xuesaitong injection, 5 [53–57] were Sanqi Tongshu capsule, 1 [58] was Xuesaitong Soft Capsule, 1 [59] was Xuesaitong dropping pill, and 1 [60] was Xueshuantong capsule. The proportion of functional independence at 3 months was reported by 1 study [36]. The total effective rate was reported by 37 studies [20–24, 26, 28, 29, 31–46, 49–61], of which 32 studies [20–22, 24, 26, 28, 29, 31, 33–46, 49–52, 54–57, 59, 61] adopted the clinical efficacy scoring standard formulated by the fourth national cerebrovascular conference, and 5 studies [23, 32, 53, 58, 60] adopted other efficacy standards. Neurological deficit scores were reported by 26 studies [21–23, 29–35, 38–41, 43, 46, 50–54, 56–58, 60, 61], of which 22 [21–23, 29–34, 38–40, 43, 46, 52–54, 56–58, 60, 61] used NIHSS and 4 [35, 41, 50, 51] used CSS. The other details are shown in Supplementary File 3. The characteristics of different PNS preparations are shown in Supplementary File 4.

### 3.3. Methodological Quality Assessment

**Figure 2:**
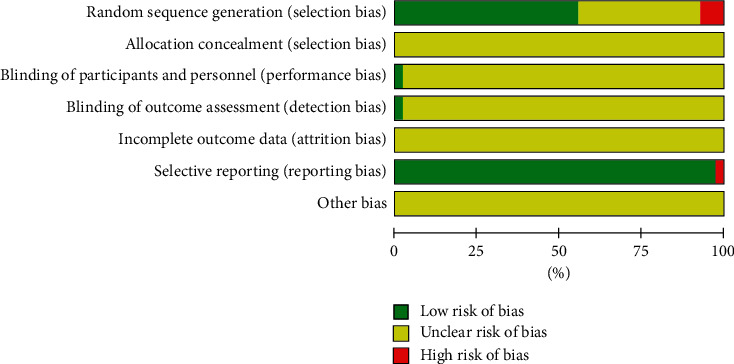
Literature quality evaluation of included studies.

We have summarized the risks of bias in the included trials in Figure 2. For “random sequence generation,” we rated twenty-four trials as having a low risk of selection bias because the authors reported a suitable randomization process, of which twenty-one trials [20–26, 28, 29, 32, 34, 35, 37–42, 51, 52, 55] used a random number table, two trials [53, 56] used systematic randomization, and one trial [30] used a lottery. Fourteen trials had an unclear risk of bias for this domain due to the lack of an adequate description of how the random sequence generation was conducted. Three trials had a high risk of bias for the domain due to the random sequence generated by admission date [60] and admission order [27, 46]. For “allocation concealment,” we considered the risk of bias to be unclear in forty-three trials, on account of the lack of reporting the allocation concealment methodology. For “blinding,” we rated one trial as having a low risk of bias because the authors explicitly reported blinding was implemented. And we judged forty-two trials as an unclear risk due to the absence of information regarding blinding of participants, personnel, and outcome assessment. For “incomplete outcome data,” we rated the risk of attrition bias as unclear in forty-three trials because the authors did not mention the loss of follow-up. For “selective reporting,” we considered forty-two trials as having a low risk of bias due to reported the preset outcomes. We judged one trial [41] as having a high risk of reporting bias because the appropriate data about the predesigned outcome was unavailable. It is not clear whether there are other biases.

### 3.4. Efficacy Outcomes

#### 3.4.1. 3-Month Functional Independence Rate

**Figure 3:**
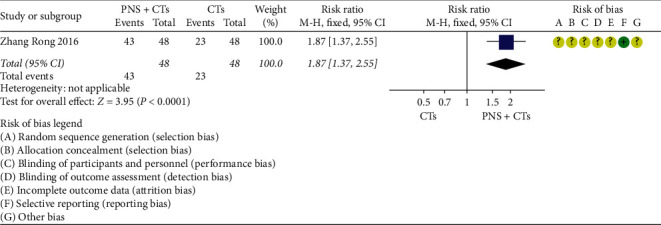
3-month functional independence rate: PNS plus CTs *vs.* CTs.

There is one study [36] that reported the functional independence rate three months after treatment. The result demonstrated that the 3-month functional independence rate for the PNS plus CTs was significantly higher than that of CTs alone (*RR* = 1.87, 95% *CI* = 1.37, to 2.55, *P* < 0.0001; Figure 3).

#### 3.4.2. NDS

**Figure 4:**
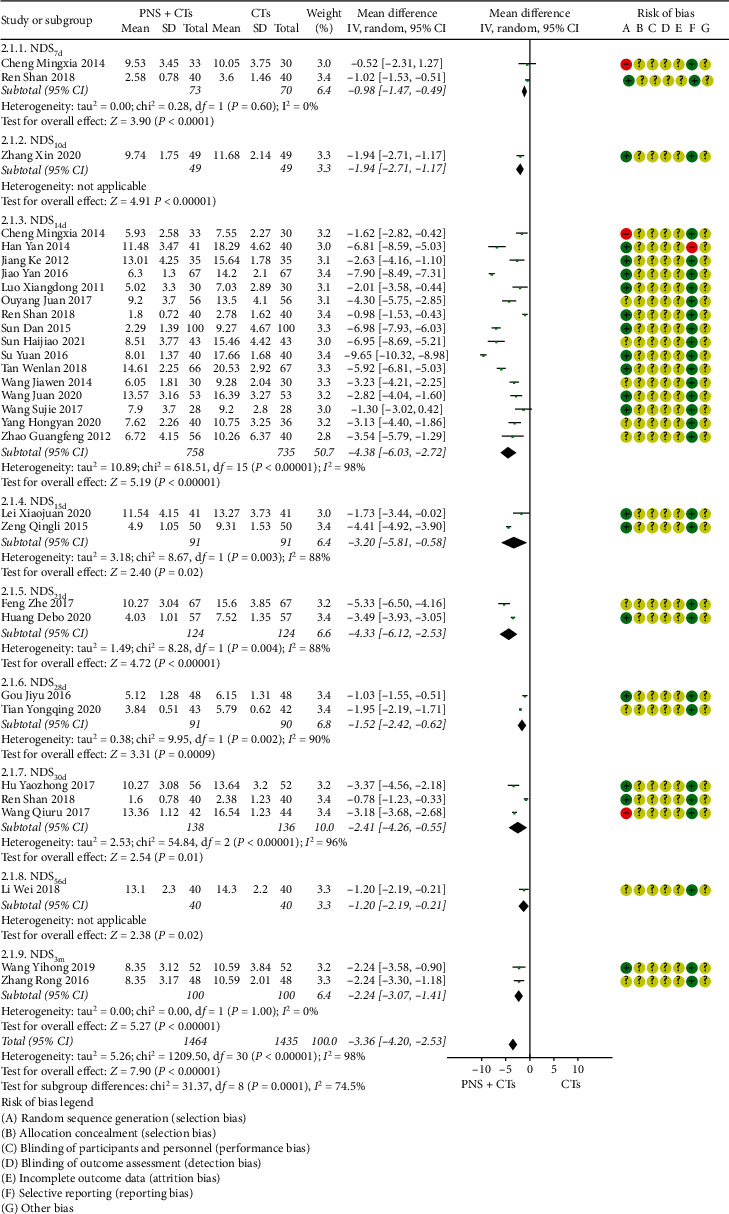
Neurologic deficit score: PNS plus CTs *vs.* CTs.

Twenty-six studies reported NDS on days 7, 10, 14, 15, 21, 28, 30, 56, and 90, respectively. Due to the substantial clinical heterogeneity and inconsistency of observation time points among studies, descriptive analysis was conducted according to the treatment time. The results are shown in Figure 4. There was no statistically significant difference in the NDS of the two studies [32, 46], while there were statistically significant differences in other studies. PNS plus CTs was related to a substantial reduction in NDS.

**Figure 5:**
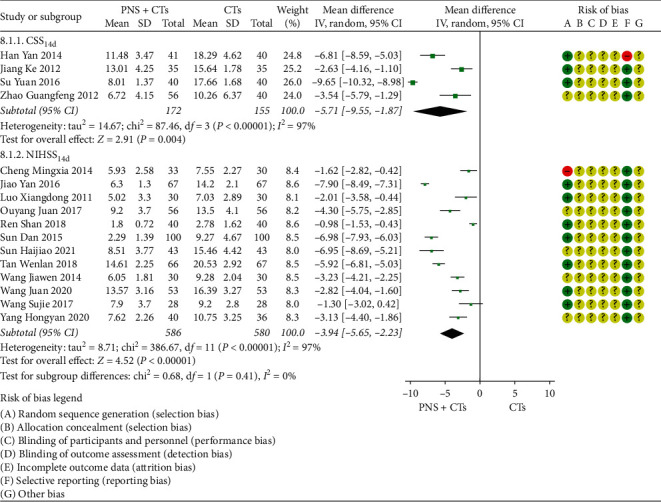
NIHSS and CSS at 14 days: PNS plus CTs *vs.* CTs.

The number of studies that observed NDS at 14 days was large; therefore, the quantitative analysis of this outcome was carried out separately. Since the different scoring standards led to significant clinical heterogeneity, which will affect the stability of the results, subgroup analysis was conducted, with the result that PNS plus CTs was associated with an evident decrease in NIHSS and CSS (*MD*_*CSS*_ = −5.71, 95% *CI* = −9.55 to −1.87, *P*=0.004; *MD*_*NIHSS*_ = −3.94, 95% *CI* = −5.65 to −2.23, *P*  < 0.00001; Figure 5). The heterogeneity between studies of two subgroups was large (*I*^2^ = 97%, *P*  < 0.00001); therefore, a random-effects model was used.

Sensitivity analysis was carried out, but the differences in the dose or duration of interventions in different studies that may cause these heterogeneities have not been found. It was suspected that different drugs of CTs resulted in heterogeneity. The results were limited by substantial heterogeneity to a certain extent.

#### 3.4.3. ADL-Barthel Score

**Figure 6:**
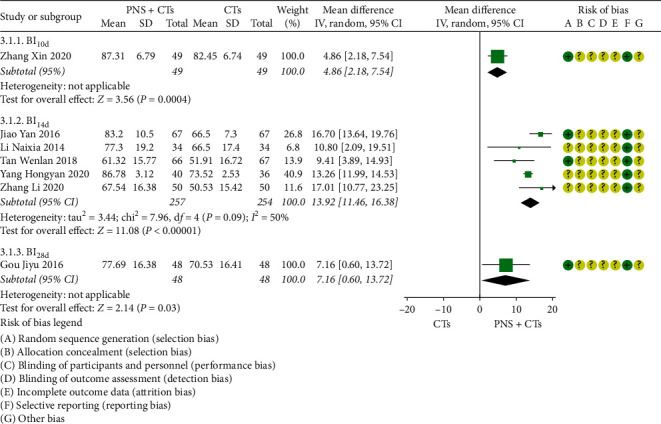
ADL-Barthel score: PNS plus CTs *vs.* CTs.

A total of seven studies [19, 23, 29, 38, 45, 56, 57] measured the changes in the ADL-Barthel score. Subgroup analysis was carried out according to different observation time points. Among the studies comparing PNS plus CTs vs. CTs alone, there was a consequential difference in the ADL-Barthel score: PNS plus CTs, as compared with CTs independently, was associated with a significant improvement in ADL-Barthel score (*MD*_*day 10 BI*_ = 4.86, 95% *CI* = 2.18, to 7.54, *P*  < 0.00001; *MD*_*day 14 BI*_ = 13.92, 95% *CI* = 11.46 to 16.38, *P*  < 0.00001; *MD*_*day 28 BI*_ = 7.16, 95% *CI* = 0.60, to 13.72, *P*  < 0.00001; Figure 6). Nevertheless, significant heterogeneity was identified among the studies (*I*^2^ = 50%, *P*=0.09), so a random-effects model was used.

**Figure 7:**
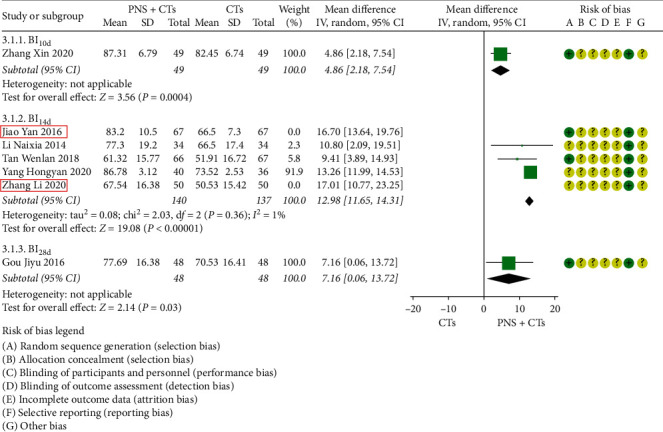
ADL-Barthel score (sensitivity analysis): PNS plus CTs *vs.* CTs.

After sensitivity analysis and careful reading of the original literature, we found that the study sites of two studies [19, 38] were quite different from those of three other studies, which were probably the major source of the heterogeneity. Tongliao and Urumqi were the sites of two studies, with dimensions of 43.6 and 43.4° north latitude, respectively, far north of the other three cities Xi'an, Shanghai, and Nanning. We considered that this was related to the influence of regional climate on blood viscosity, which will be further explored in the future. The two studies were removed, and the other three studies were pooled alone (*MD* = 12.98, 95% *CI* = 11.65 to 14.31, *P* < 0.00001; Figure 7). Heterogeneity between the three studies was insignificant (*I*^*2*^ = 1%, *P*=0.36).

#### 3.4.4. ORR

**Figure 8:**
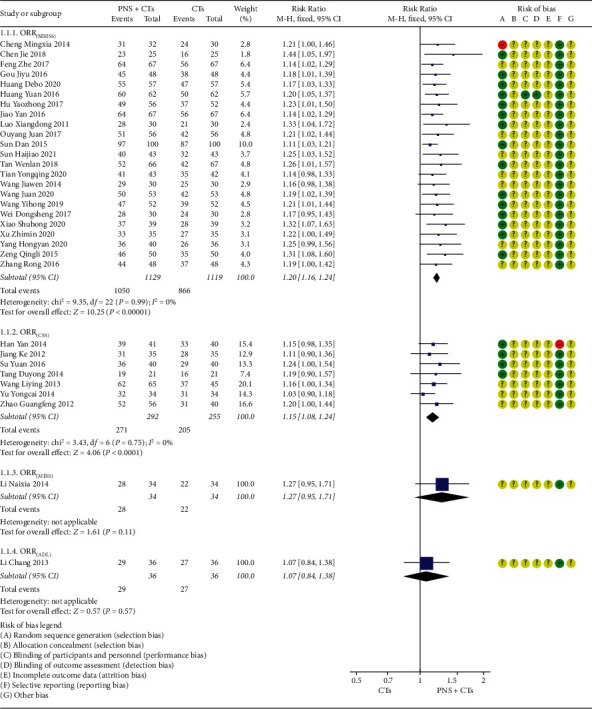
ORR: PNS plus CTs *vs.* CTs.

There are thirty-seven studies that reported the overall response rate. Among them, thirty-two studies adopted the clinical efficacy scoring standard developed by the fourth national cerebrovascular conference in China in 1998, in which there are also four evaluation criteria for functional recovery. Subgroup analysis was performed according to different evaluation criteria. The heterogeneities of the NIHSS group and CSS group were not obvious (*I*^2^_*NIHSS*_ = 0%, *P*=0.99; *I*^2^*CSS* = 0%, *P*=0.75), so the fixed-effects model was used. The results demonstrated that the ORR for the PNS plus CTs was significantly higher than that of CTs alone (*RR*_*NIHSS*_ = 1.20, 95% *CI* = 1.16, to 1.24, *P* < 0.00001; *RR*_*CSS*_ = 1.15, 95% *CI* = 1.08, to 1.24, *P* < 0.0001; Figure 8). There was no significant difference in the other two studies [45, 59] with the score of MESS and ADL.

#### 3.4.5. Hemorheology

**Figure 9:**
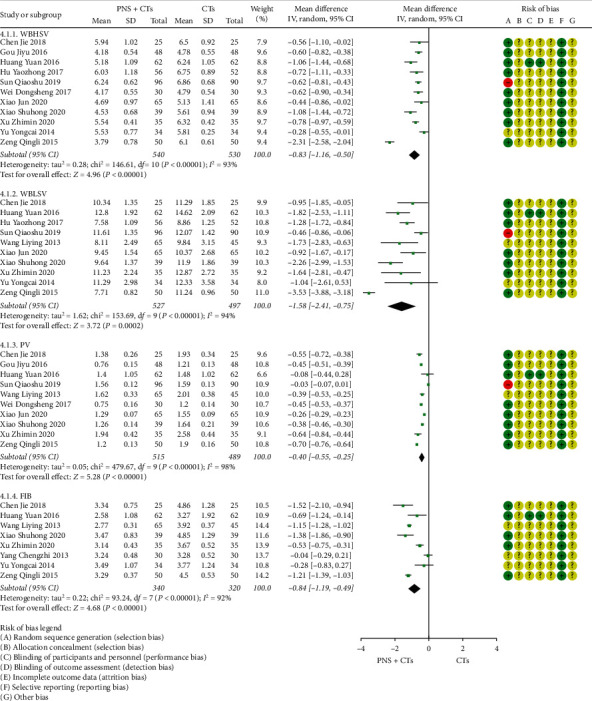
Hemorheology: PNS plus CTs *vs.* CTs.

A total of fourteen studies reported hemorheological parameters, including eleven [24, 25, 27, 28, 34, 37, 39, 44, 55, 56] for whole blood high shear viscosity (WBHSV), ten [20, 24, 25, 27, 28, 34, 37, 39, 44, 49] for whole blood low shear viscosity (WBLSV), ten [20, 24, 25, 27, 28, 37, 39, 49, 55, 56] for plasma viscosity (PV), and eight [20, 24, 28, 37, 39, 44, 48, 49] for fibrinogen (FIB). The heterogeneities between the studies were large (*I*^2^_*WBHSV*_ = 93%, *P* < 0.00001; *I*^2^_*WBHSV*_ = 94%, *P* < 0.00001; *I*^2^_*PV*_ = 98%, *P* < 0.00001; *I*^2^_*FIB*_ = 92%, *P* < 0.00001), and the heterogeneities still existed after subgroup analysis according to the course of treatment, dose, and dosage form. No other obvious sources of heterogeneities were found in sensitivity analysis, so descriptive analysis was used. See Figure 9 for results. Except for four studies [27, 37, 44, 48], the differences were statistically significant. The results showed that PNS plus CTs can effectively improve the hemorheology of patients with acute cerebral infarction.

#### 3.4.6. MPAR

**Figure 10:**
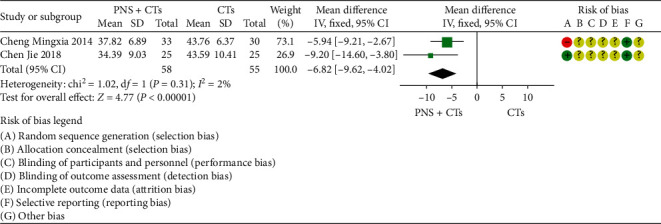
MPAR: PNS plus CTs *vs.* CTs.

Two studies [28, 46] were assessed for the MPAR after patients had been treated. The results demonstrated that PNS plus CTs showed a weighty decrease on the MPAR compared with CTs alone (*MD* = −6.82, 95% *CI* = −9.62 to −4.02, *P* < 0.00001; Figure 10). The heterogeneity among the two studies was insignificant (*I*^*2*^ = 2%, *P*=0.31), and a fixed-effects model was used.

#### 3.4.7. Platelet Parameters

**Figure 11:**
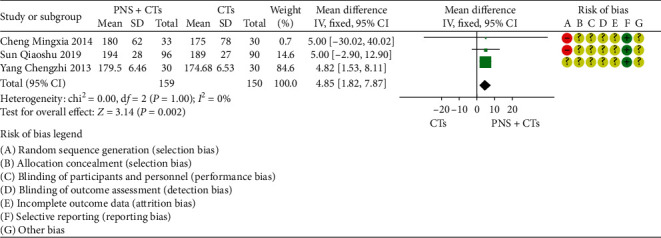
PLT: PNS plus CTs *vs.* CTs.

**Figure 12:**
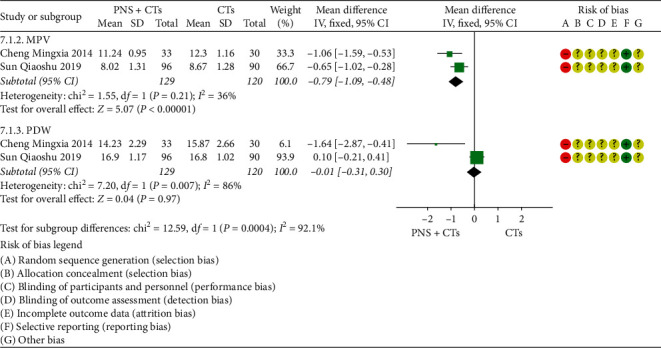
MPV and PDW: PNS plus CTs *vs.* CTs.

A total of four studies measured platelet parameters, including four [27, 39, 46, 48] for platelet count (PLT), two [27, 46] for mean platelet volume (MPV), and two [27, 46] for platelet distribution width (PDW). Since the result of one study [39] was contrary to the conclusion, PLT was finally included in three studies. The results demonstrated that PNS plus CTs was better than CTs in improving PLT (*MD*_*PLT*_ = 4.85, 95% *CI* = 1.82 to 7.84, *P*=0.002; Figure 11) and reducing MPV (*MD*_*MPV*_ = −0.79, 95% *CI* = −1.09 to −0.48, *P* < 0.00001; Figure 12). The heterogeneity among studies was insignificant (*I*^2^_*PLT*_ = 0%, *P*=1.00; *I*^2^_*MPV*_ = 36%, *P*=0.21), and a fixed-effects model was used. In addition, the differences were not statistically significant in changing PDW (*MD*_*PDW*_ = −0.01, 95% *CI* = −0.31 to 0.30, *P*=0.97; Figure 12).

#### 3.4.8. CD62P

**Figure 13:**
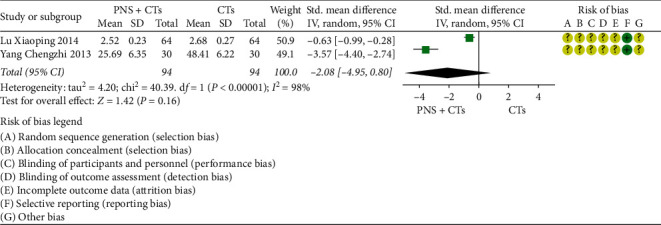
Platelet membrane CD62P: PNS plus CTs *vs.* CTs.

**Figure 14:**
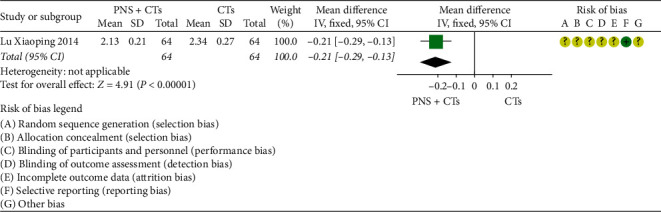
Serum CD62P: PNS plus CTs *vs.* CTs.

Two studies [47, 48] were assessed for the CD62P, one of which observed serum CD62P and platelet membrane CD62P, and the other only observed platelet membrane CD62P. The results of the two studies were positive, but the combined confidence interval included zero (*SMD* = −2.08, 95% *CI* = −4.95 to 0.80, *P*=0.16; Figure 13). The heterogeneity between the two studies was significant (*I*^*2*^ = 98%, *P* < 0.00001), so a random-effects model was used. After rereading the full text to verify the extracted data and find the reason for the heterogeneity, we considered the heterogeneity resulting from different units. Although both studies indicated that the tool for measuring platelet membrane CD62P was flow cytometry, the data results showed that the observation value of one study was concentration and the observation value of the other study was expression rate. No significant clinical heterogeneity was found between the two studies. Since the units of the same outcome are different and cannot be converted, *SMD* was used for consolidation. In addition, the serum CD62P observed in one study showed that PNS + CTs decreased more than CTs (*MD* = −0.21, 95% *CI* = −0.29 to −0.13, *P* < 0.00001; Figure 14).

#### 3.4.9. Coagulation Function

**Figure 15:**
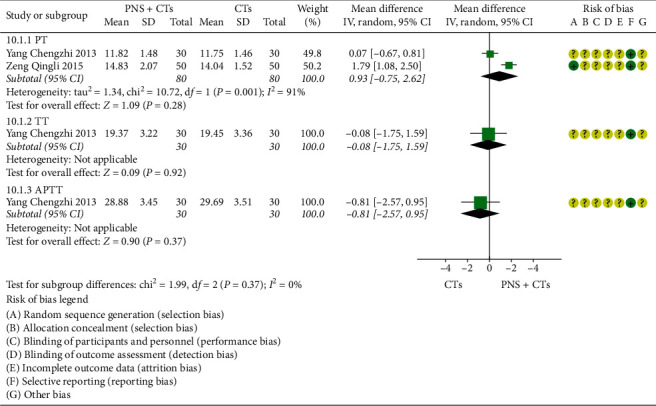
Coagulation function: PNS plus CTs *vs.* CTs.

Two studies [39, 48] assessed the coagulation function. The heterogeneity among the two studies was significant (*I*^2^ = 91%, *P*=0.001), and a random-effects model was used. In the prothrombin time (PT), activated partial thromboplastin time (APTT), and thrombin time (TT), there was no statistical difference between the two groups (*MD*_*PT*_ = 0.93, 95% *CI* = −0.75 to 2.62, *P*=0.28; *MD*_*TT*_ = −0.08, 95% *CI* = −1.75 to 1.59, *P*=0.92; *MD*_*APTT*_ = −0.81, 95% *CI* = −2.57 to 0.95, *P*=0.37; Figure 15).

After sensitivity analysis and careful reading of the original literature, we found that the course of treatment between the two studies was different, which might be the major source of the heterogeneity.

### 3.5. Safety Outcomes

#### 3.5.1. Incidence of Adverse Reactions

**Figure 16:**
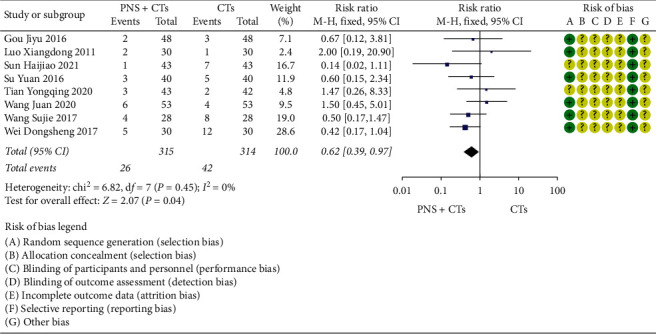
Incidence of adverse reactions: PNS plus CTs *vs.* CTs.

Eight studies [21, 32, 35, 52, 54–56, 61] recorded the incidence of adverse reactions. Incidence of adverse reactions occurred in 26 out of 315 patients (8.3%) who received PNS plus CTs and 42 out of 314 patients (13.4%) who received CTs alone. The heterogeneity among eight studies was insignificant (*I*^*2*^ = 0.0%, *P*=0.45), and a fixed-effects model was used. The incidence of adverse reactions of the experimental group was lower than that of the control group (*RR* = 0.62, 95% *CI* = 0.39 to 0.97, *P*=0.04; Figure 16).

#### 3.5.2. Adverse Reactions

Fifteen studies [21, 29, 31, 32, 34, 35, 43, 44, 52, 54–58, 61] reported adverse reactions. Among them, six studies [29, 31, 34, 43, 44, 58] reported no adverse reaction in both groups, and the other studies reported adverse reactions in two groups including gastrointestinal reactions, skin rashes, abnormal liver function, palpitation, infusion reaction, and other unexplained adverse reactions. No participants discontinued the study drug due to adverse reactions.

### 3.6. Publication Bias

**Figure 17:**
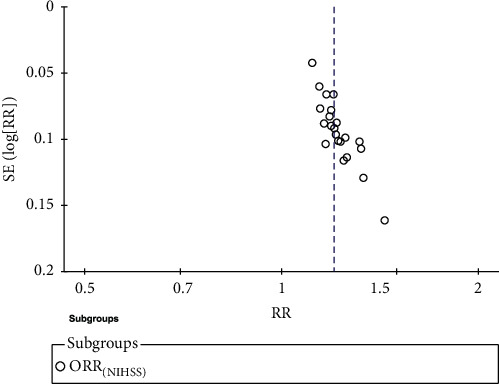
The funnel diagram of PNS plus CTs and CTs to compare ORRNIHSS.

The ORRNIHSS of twenty-three studies was evaluated by the funnel chart, and the results showed that the left-right asymmetry may be related to the low methodological quality and unpublished negative results of the included studies, as shown in Figure 17.

### 3.7. GRADE Assessment

The GRADE system was used to assess the level of evidence for the twelve outcomes, which indicated low or very low quality with serious methodological problems, a heterogeneity problem, and a small sample problem. The GRADE evidence profiles are shown in Supplementary File 5.

## 4. Discussion

### 4.1. Summary of Evidence

In the current systematic review, we evaluated the efficacy of Panax Notoginseng Saponins (PNS) including four types of Chinese medicine injection and four types of oral Chinese patent medicine to treat patients with AIS. We conducted a comprehensive literature search and identified 43 RCTs (4170 participants) for analysis. Compared with CTs, PNS plus CTs was more effective in the treatment of patients with AIS, in increasing the proportion of patients with independent function after 3 months (only one small sample study), improving neurological function, and restoring activities of daily living. Over the past few years, pharmacological experiments found that PNS can increase the blood oxygen supply of ischemic tissue by maintaining the physiological function of mitochondria [62], promoting the proliferation of vascular endothelial cells [63], promoting angiogenesis [64], and improving hemorheology [65] in the treatment of cerebral ischemia.

For the laboratory outcomes, the results showed positive effects of PNS on improving WBHSV, WBLSV, PV, and FIB. In the aspect of antiplatelet effects, PNS can effectively reduce MPAR and MPV and increase PLT. However, there is insufficient evidence for PNS to inhibit the expression of CD62P and improve coagulation function. In surviving AIS patients, the reduction in platelet aggregation (PA) was accompanied by improvements in the clinical condition, whereas the negative dynamic of PA was recorded in deceased patients [66]. And Tsuyoshi Uesugi's study also found that the recurrence rate of ischemic stroke in patients with inhibition of PA after antiplatelet therapy was significantly lower than that in patients with unchanged PA [67]. Although platelet function testing may be of guiding significance in drug therapy to improve the prognosis of AIS, a study had shown that platelet function-guided modification in antiplatelet therapy after AIS was associated with significantly higher rates of adverse clinical outcomes [68]. The latest systematic review found that MPV was significantly higher and PLT was significantly lower in patients with ischemic stroke [69], so they may be used as markers to predict the recurrence of ischemic stroke. Nevertheless, Irene Ciancarelli's study provided that MPV was not a marker of neurologic deficit and disability or of stroke recovery including motor performance and functional independence and cannot be used to evaluate the prognosis of AIS [70].

For the safety outcomes, the results showed that the incidence of adverse reactions in the PNS group was lower than that in the CT group. The adverse reactions of the experimental group were mainly mild gastrointestinal discomfort and rash, which suggested that PNS should be used carefully in patients with chronic gastric disease and allergy history.

### 4.2. Strengths and Limitations

Compared with the previous reviews, the current systematic review is comprehensive and included 43 trials, which provides relatively complete and up-to-date evidence on the use of PNS as adjunct therapies for AIS. We used an evidence-based medicine approach to critically review the existing evidence from previous RCTs, and we found a better effect of PNS for independent function, platelet parameters, and MPAR. In addition, we applied GRADE criteria to determine the certainty in the estimate of effect for important outcomes.

There are some limitations to our review that need to be acknowledged. Firstly, in the real world, various drugs are commonly used in the treatment of AIS. Although we strictly limited the drug category of CTs in the eligibility criteria, in our review, most of the trials did not mention the specific therapeutic regimen, which resulted in inevitable clinical heterogeneity to a certain extent. Furthermore, excessive statistical heterogeneity came to our attention in some of the comparisons. However, we cannot identify the source of heterogeneity through the data and information provided. The quality of the included trials is generally poor in random sequence generation and blind design, which is a common problem in the current situation of clinical trials of TCM [71]. In addition, the insufficient sample size of included studies in some comparisons affected the reliability of the results. Lastly, as some randomized, double-blind, controlled large-sample clinical trials are ongoing, such as the RCT of Xuesaitong soft capsules treating patients with AIS conducted by Xuanwu Hospital Capital Medical University, evidence from the current analysis is incomplete, and further updates are expected to complement the results of this systematic review.

### 4.3. Implications for Future Research

Long-term outcomes, such as 3-month favorable functional outcome, should be chosen as the primary outcome, instead of using intermediate outcomes to substitute for endpoint outcomes as many clinical trials of TCM [72]. The measurement time of various outcomes should be standardized [73] to ensure the data merging between different studies. NIHSS score is suggested to be used in evaluating the neurological deficit uniformly, in order to avoid the heterogeneity caused by different standards. It is hoped that more attention will be paid to the occurrence of bleeding events during the treatment of AIS, since the combination of antiplatelet drugs, anticoagulants, and PNS makes it difficult to evaluate the bidirectional regulation function only through laboratory indicators such as MPAR, MPV, and PT.

Future researchers are urged to design experiments based on a rigorous methodology, including appropriate sample sizes and adequate follow-up with long-term duration, and the standardized report will be carried out according to the guidelines of SPIRIT-TCM Extension 2018 [74] and CONSORT-CHM formulas 2017 [75]. In terms of safety analysis, researchers must assess whether the adverse events are related to drug use. And economic analysis should be considered to guide practices.

## 5. Conclusion

We found that PNS combined with CTs has a certain effect on the treatment of AIS. However, due to the small number of studies and the high risks of bias, the above evidence is low to very low and the safety remains uncertain. In the future, more strong evidence for clinical practice requires large-scale and high-quality RCT.

## Data Availability

The data used in the article are obtained from public databases. The processes including the literature, data extraction, and calculation are all described in the article. If necessary, the first author LDW (liudingwang97@163.com) can be contacted to obtain data.

## Supplementary Materials

Supplementary Materials
*Supplementary File 1*. Table S1 containing search strategy. *Supplementary File 2*. Table S2 containing the list of excluded reports. *Supplementary File 3*. Table S3 containing the basic characteristics of included studies. *Supplementary File 4*. Table S4 containing the basic characteristics of PNS preparations. *Supplementary File 5*. Table S5 containing a GRADE summary of outcomes. *Supplementary File 6*. PRISMA 2020 checklist. *Supplementary File 7.* Research protocol.
