# Supplementary material for: Systematic Review and Meta-Analysis on Randomized Controlled Trials on Efficacy and Safety of Panax Notoginseng Saponins in Treatment of Acute Ischemic Stroke
Source: Evid Based Complement Alternat Med. 2021 Jul 9;2021:4694076. doi: 10.1155/2021/4694076 (PMC8289597; doi:10.1155/2021/4694076)
Supplement: Supplementary Materials — Supplementary File 1. Table S1 containing search strategy. Supplementary File 2. Table S2 containing the list of excluded reports. Supplementary File 3. Table S3 containing the basic characteristics of included studies. Supplementary File 4. Table S4 containing the basic characteristics of PNS preparations. Supplementary File 5. Table S5 containing a GRADE summary of outcomes. Supplementary File 6. PRISMA 2020 checklist. Supplementary File 7. Research protocol. [file 4694076.f1.zip › 4694076.f1/Supplementary files 1.docx]

| **Table S1. The search strategy for PubMed** | |
| --- | --- |
| **Number** | **Search terms** |
| #1 | Cerebral infarction[MeSH Terms] |
| #2 | Cerebral infarction[Title/Abstract] |
| #3 | (((((((((((((((((((((((Cerebral Infarctions[Title/Abstract]) OR (Infarctions, Cerebral[Title/Abstract])) OR (Infarction, Cerebral[Title/Abstract])) OR (Cerebral Infarct[Title/Abstract])) OR (Cerebral Infarcts[Title/Abstract])) OR (Infarct, Cerebral[Title/Abstract])) OR (Cerebral Infarction, Left Hemisphere[Title/Abstract])) OR (Left Hemisphere, Infarction, Cerebral[Title/Abstract])) OR (Infarction, Left Hemisphere, Cerebral[Title/Abstract])) OR (Left Hemisphere, Cerebral Infarction[Title/Abstract])) OR (Cerebral, Left Hemisphere, Infarction[Title/Abstract])) OR (Infarction, Cerebral, Left Hemisphere[Title/Abstract])) OR (Subcortical Infarction[Title/Abstract])) OR (Infarction, Subcortical[Title/Abstract])) OR (Infarctions, Subcortical[Title/Abstract])) OR (Subcortical Infarctions[Title/Abstract])) OR (Posterior Choroidal Artery Infarction[Title/Abstract])) OR (Anterior Choroidal Artery Infarction[Title/Abstract])) OR (Cerebral Infarction, Right Hemisphere[Title/Abstract])) OR (Right Hemisphere, Cerebral Infarction[Title/Abstract])) OR (Infarction, Right Hemisphere, Cerebral[Title/Abstract])) OR (Right Hemisphere, Infarction, Cerebral[Title/Abstract])) OR (Cerebral, Right Hemisphere, Infarction[Title/Abstract])) OR (Infarction, Cerebral, Right Hemisphere[Title/Abstract]) |
| #4 | #1 OR #2 OR #3 |
| #5 | stroke[MeSH Terms] |
| #6 | stroke[Title/Abstract] |
| #7 | ((((((((((((((((((((((((((Strokes[Title/Abstract]) OR (Cerebrovascular Accident[Title/Abstract])) OR (CVA (Cerebrovascular Accident)[Title/Abstract])) OR (CVAs (Cerebrovascular Accident)[Title/Abstract])) OR (Cerebrovascular Apoplexy[Title/Abstract])) OR (Apoplexy, Cerebrovascular[Title/Abstract])) OR (Vascular Accident, Brain[Title/Abstract])) OR (Brain Vascular Accident[Title/Abstract])) OR (Brain Vascular Accidents[Title/Abstract])) OR (Brain Vascular Accidents[Title/Abstract])) OR (Cerebrovascular Stroke[Title/Abstract])) OR (Cerebrovascular Strokes[Title/Abstract])) OR (Stroke, Cerebrovascular[Title/Abstract])) OR (Strokes, Cerebrovascular[Title/Abstract])) OR (Apoplexy[Title/Abstract])) OR (Cerebral Stroke[Title/Abstract])) OR (Cerebral Strokes[Title/Abstract])) OR (Stroke, Cerebral[Title/Abstract])) OR (Strokes, Cerebral[Title/Abstract])) OR (Stroke, Acute[Title/Abstract])) OR (Acute Stroke[Title/Abstract])) OR (Acute Strokes[Title/Abstract])) OR (Strokes, Acute[Title/Abstract])) OR (Cerebrovascular Accident, Acute[Title/Abstract])) OR (Acute Cerebrovascular Accident[Title/Abstract])) OR (Acute Cerebrovascular Accidents[Title/Abstract])) OR (Cerebrovascular Accidents, Acute[Title/Abstract]) |
| #8 | #5 OR #6 OR #7 |
| #9 | #4 OR #8 |
| #10 | (((((((((Panax notoginseng extract[Title/Abstract]) OR (Xueshuantong[Title/Abstract])) OR (Xue shuan tong[Title/Abstract])) OR (Xuesetong[Title/Abstract])) OR (xuesaitong[Title/Abstract])) OR (xuesetong injection[Title/Abstract])) OR (xue sai tong[Title/Abstract])) OR (sanqi[Title/Abstract])) OR (Sanqi Tongshu capsule[Title/Abstract])) OR (Lulutong[Title/Abstract]) |
| #11 | #9 AND #10 |
| **The search strategy for Embase** | |
| **Number** | **Search terms** |
| #1 | 'cerebral infarction'/exp |
| #2 | 'Cerebral Infarctions':ti,ab,kw OR 'Infarctions, Cerebral':ti,ab,kw OR 'Infarction, Cerebral':ti,ab,kw OR 'Cerebral Infarct':ti,ab,kw OR 'Cerebral Infarcts':ti,ab,kw OR ' Infarct, Cerebral':ti,ab,kw OR 'Infarcts, Cerebral':ti,ab,kw OR 'Cerebral Infarction, Left Hemisphere':ti,ab,kw OR 'Left Hemisphere, Infarction, Cerebral':ti,ab,kw OR 'Infarction':ti,ab,kw OR 'Left Hemisphere, Cerebral Left Hemisphere, Cerebral Infarction':ti,ab,kw OR 'Cerebral, Left Hemisphere, Infarction':ti,ab,kw OR 'Infarction, Cerebral, Left Hemisphere':ti,ab,kw OR 'Subcortical Infarction':ti,ab,kw OR 'Infarction, Subcortical':ti,ab,kw OR 'Infarctions, Subcortical':ti,ab,kw OR 'Subcortical Infarctions':ti,ab,kw OR 'Posterior Choroidal Artery Infarction':ti,ab,kw OR 'Anterior Choroidal Artery Infarction':ti,ab,kw OR 'Cerebral Infarction, Right Hemisphere':ti,ab,kw OR 'Right Hemisphere, Cerebral Infarction':ti,ab,kw OR 'Infarction, Right Hemisphere, Cerebral':ti,ab,kw OR 'Right Hemisphere, Infarction, Cerebral':ti,ab,kw OR 'Cerebral, Right Hemisphere, Infarction':ti,ab,kw OR 'Infarction, Cerebral, Right Hemisphere':ti,ab,kw |
| #3 | 'Stroke'/exp |
| #4 | 'Cerebrovascular Accident':ti,ab,kw OR 'Cerebrovascular Accidents':ti,ab,kw OR ' CVA (Cerebrovascular Accident)':ti,ab,kw OR 'CVAs (Cerebrovascular Accident)':ti,ab,kw OR 'Cerebrovascular Apoplexy':ti,ab,kw OR 'Apoplexy, Cerebrovascular':ti,ab,kw OR 'Vascular Accident, Brain':ti,ab,kw OR 'Brain Vascular Accident':ti,ab,kw OR 'Brain Vascular Accidents':ti,ab,kw OR 'Vascular Accidents, Brain':ti,ab,kw OR 'Cerebrovascular Stroke':ti,ab,kw OR 'Cerebrovascular Strokes':ti,ab,kw OR 'Stroke, Cerebrovascular':ti,ab,kw OR 'Strokes, Cerebrovascular':ti,ab,kw OR 'Apoplexy':ti,ab,kw OR 'Cerebral Stroke':ti,ab,kw OR 'Cerebral Strokes':ti,ab,kw OR 'Stroke, Cerebral':ti,ab,kw OR 'Strokes, Cerebral':ti,ab,kw OR 'Stroke, Acute':ti,ab,kw OR 'Acute Stroke':ti,ab,kw OR 'Acute Strokes':ti,ab,kw OR 'Strokes, Acute':ti,ab,kw OR 'Cerebrovascular Accident, Acute':ti,ab,kw OR 'Acute Cerebrovascular Accident':ti,ab,kw OR 'Acute Cerebrovascular Accidents':ti,ab,kw OR 'Cerebrovascular Accidents, Acute':ti,ab,kw |
| #5 | 'Panax notoginseng extract':ti,ab,kw OR Xueshuantong:ti,ab,kw OR Xue shuan tong:ti,ab,kw OR Xuesetong:ti,ab,kw OR xuesaitong:ti,ab,kw OR xuesetong injection:ti,ab,kw OR xue sai tong:ti,ab,kw OR sanqi:ti,ab,kw OR Sanqi Tongshu capsule:ti,ab,kw OR Lulutong:ti,ab |
| #6 | #1 OR #2 OR #3 OR #4 |
| #7 | #5 AND #6 |
| **The search strategy for Cochrane Library** | |
| **Number** | **Search terms** |
| #1 | MeSH descriptor: [Cerebral Infarction] explode all trees |
| #2 | (Cerebral Infarctions):ti,ab,kw OR (Infarctions, Cerebral):ti,ab,kw OR (Infarction, Cerebral):ti,ab,kw OR (Cerebral Infarct):ti,ab,kw OR (Cerebral Infarcts):ti,ab,kw |
| #3 | (Infarct, Cerebral):ti,ab,kw OR (Infarcts, Cerebral):ti,ab,kw OR (Cerebral Infarction, Left Hemisphere):ti,ab,kw OR (Left Hemisphere, Infarction, Cerebral):ti,ab,kw OR (Infarction, Left Hemisphere, Cerebral):ti,ab,kw |
| #4 | (Left Hemisphere, Cerebral Infarction):ti,ab,kw OR (Cerebral, Left Hemisphere, Infarction):ti,ab,kw OR (Subcortical Infarctions):ti,ab,kw OR (Infarctions, Subcortical):ti,ab,kw OR (Infarction, Subcortical):ti,ab,kw |
| #5 | (Subcortical Infarction):ti,ab,kw OR (Infarction, Cerebral, Left Hemisphere):ti,ab,kw OR (Right Hemisphere, Cerebral Infarction):ti,ab,kw OR (Cerebral Infarction, Right Hemisphere):ti,ab,kw OR (Anterior Choroidal Artery Infarction):ti,ab,kw |
| #6 | (Posterior Choroidal Artery Infarction):ti,ab,kw OR (Infarction, Cerebral, Right Hemisphere):ti,ab,kw OR (Cerebral, Right Hemisphere, Infarction):ti,ab,kw OR (Right Hemisphere, Infarction, Cerebral):ti,ab,kw OR (Infarction, Right Hemisphere, Cerebral):ti,ab,kw |
| #7 | (Panax notoginseng extract):ti,ab,kw OR (Xueshuantong):ti,ab,kw OR (Xue shuan tong):ti,ab,kw OR (Xuesetong):ti,ab,kw OR (xuesaitong):ti,ab,kw OR (xuesetong injection):ti,ab,kw OR (xue sai tong):ti,ab,kw OR (sanqi):ti,ab,kw OR (Sanqi Tongshu capsule):ti,ab,kw OR (Lulutong):ti,ab,kw |
| #8 | #1 OR #2 OR #3 OR #4 OR #5 OR #6 |
| #9 | #7 AND #8 |
| **The search strategy for CNKI** | |
| (SU = '缺血性脑卒中' OR SU = '缺血性卒中' OR SU = '缺血性脑中风' OR SU = '缺血性中风' OR SU = '缺血性脑血管病' OR SU = '脑梗死' OR SU = '脑梗' OR SU = '脑栓塞' OR SU = '脑缺血' OR SU = '脑卒中' OR SU = '脑梗塞' OR SU = '中风' OR SU = '卒中' OR SU = '小脑梗塞' OR SU = '脑干梗塞' OR SU = '脑中风' OR SU = '脑血管意外' OR SU = '脑血栓') AND (SU= '三七' OR SU= '血塞通' OR SU= '血栓通' OR SU= '路路通') AND(SU = '急性' OR SU = '超早期' OR SU = '进展性' OR SU = '恶化性' OR SU = '早期神经系统功能恶化' OR SU = '超急性期') | |
| **The search strategy for WanFang** | |
| 主题: ("缺血性脑卒中"+"缺血性卒中"+"缺血性脑中风"+"缺血性中风"+"缺血性脑血管病"+"脑梗死"+"脑梗"+"脑栓塞"+"脑缺血"+"脑卒中"+"脑梗塞"+"中风"+"卒中"+"小脑梗塞"+"脑干梗塞"+"脑中风"+"脑血管意外"+"脑血栓") and 主题: ("三七"+"血塞通"+"血栓通"+"路路通")and 主题：("急性"+"超早期"+"进展性"+"恶化性"+"早期神经系统功能恶化"+"超急性期") | |
| **The search strategy for VIP** | |
| ((M=缺血性脑卒中 OR 缺血性卒中 OR 缺血性脑中风 OR 缺血性中风 OR 缺血性脑血管病 OR 脑梗死 OR 脑梗 OR 脑栓塞 OR 脑缺血 OR 脑卒中 OR 脑梗塞 OR 中风 OR 卒中 OR 小脑梗塞 OR 脑干梗塞 OR 脑中风 OR 脑血管意外 OR 脑血栓) OR (R=缺血性脑卒中 OR 缺血性卒中 OR 缺血性脑中风 OR 缺血性中风 OR 缺血性脑血管病 OR 脑梗死 OR 脑梗 OR 脑栓塞 OR 脑缺血 OR 脑卒中 OR 脑梗塞 OR 中风 OR 卒中 OR 小脑梗塞 OR 脑干梗塞 OR 脑中风 OR 脑血管意外 OR 脑血栓)) AND ((M=三七 OR 血塞通 OR 血栓通 OR 路路通) OR (R=三七 OR 血塞通 OR 血栓通 OR 路路通)) AND ((M=急性 OR 超早期 OR 进展性 OR 恶化性 OR 早期神经系统功能恶化 OR 超急性期) OR (R=急性 OR 超早期 OR 进展性 OR 恶化性 OR 早期神经系统功能恶化 OR 超急性期)) | |
| **The search strategy for CBM** | |
| #1 | "缺血性脑卒中"[常用字段:智能] OR "缺血性卒中"[常用字段:智能] OR "缺血性脑中风"[常用字段:智能] OR "缺血性中风"[常用字段:智能] OR "缺血性脑血管病"[常用字段:智能] OR "脑梗死"[常用字段:智能] OR "脑梗"[常用字段:智能] OR "脑栓塞"[常用字段:智能] OR "脑缺血"[常用字段:智能] |
| #2 | "脑卒中"[常用字段:智能] OR "脑梗塞"[常用字段:智能] OR "中风"[常用字段:智能] OR "卒中"[常用字段:智能] OR "小脑梗塞"[常用字段:智能] OR "脑干梗塞"[常用字段:智能] OR "脑中风" |
| #3 | "急性"[常用字段:智能] OR "超早期"[常用字段:智能] OR "进展性"[常用字段:智能] OR "恶化性"[常用字段:智能] OR "早期神经系统功能恶化"[常用字段:智能] OR "超急性期"[常用字段:智能] |
| #4 | "三七"[常用字段:智能] OR "血塞通"[常用字段:智能] OR "血栓通"[常用字段:智能] OR "路路通"[常用字段:智能] |
| #5 | #1 OR #2 |
| #6 | #3 AND #4 AND #5 |
