# Supplementary material for: Systematic Review and Meta-Analysis on Randomized Controlled Trials on Efficacy and Safety of Panax Notoginseng Saponins in Treatment of Acute Ischemic Stroke
Source: Evid Based Complement Alternat Med. 2021 Jul 9;2021:4694076. doi: 10.1155/2021/4694076 (PMC8289597; doi:10.1155/2021/4694076)
Supplement: Supplementary Materials — Supplementary File 1. Table S1 containing search strategy. Supplementary File 2. Table S2 containing the list of excluded reports. Supplementary File 3. Table S3 containing the basic characteristics of included studies. Supplementary File 4. Table S4 containing the basic characteristics of PNS preparations. Supplementary File 5. Table S5 containing a GRADE summary of outcomes. Supplementary File 6. PRISMA 2020 checklist. Supplementary File 7. Research protocol. [file 4694076.f1.zip › 4694076.f1/Supplementary files 2.docx]

| Reports excluded | Reason |
| --- | --- |
| Zhang Wenjie 2019 [1] | Unclear outcome |
| Mo Xiuying 2019 [2] | Unclear outcome |
| Xiang Fei 2014 [3] | Unclear outcome |
| Gao Zhengju 2011 [4] | Unclear outcome |
| Lai Jianxing 2008 [5] | Unclear outcome |
| Liu Xindong 2019 [6] | Wrong data |
| Gu Yumei 2017 [7] | Wrong data |
| Duan Shixiang 2012 [8] | Incorrect interventions |
| Ma Junhua 2010 [9] | Incorrect interventions |

**References**

1. W. Zhang and D. Li, “Clinical study on the treatment of acute ischemic stroke with thrombolytic injection,” *Chinese Journal Of integrative Medicine On Cardio-/Cerebrovascuiar Disease*, vol. 17, no. 14, pp. 2235-2237, 2019.

[2] X. Mo, X. Wang and M. Chen, “Clinical observation of alteplase combined with Xueshuantong injection in the treatment of acute cerebral infarction,” *China's Naturopathy*, vol. 27, no. 11, pp. 62-63, 2019.

[3] F. Xiang, X. Zhang and L. Jiang, “The effect of thrombotong assisted conventional western medicine on hs CRP, IL-6 and D-dimer in patients with acute cerebral infarction,” *Journal of Emergency in Traditional Chinese Medicine*, vol. 23, no. 12, pp. 2287-2289, 2014.

[4] Z. Gao, “Clinical study of Xuesaitong and ozagrel sodium in the treatment of acute cerebral infarction,” *Journal of Frontiers of Medicine*, vol. 01, no. 19, pp. 79-80, 2011.

[5] J. Lai and B. Wu, “Therapeutic effect of Xueshuantong on cerebral infarction,” *Chinese Journal of Current Practical Medicine*, vol. 7, no. 9, pp. 13-14, 2008.

[6] X. Liu, Li Zhang, L. Yang, et al., “Effect and mechanism of Xueshuantong combined with alteplase on neurological function in patients with acute cerebral infarction,” *Chinese Journal of Hospital Pharmacy*, vol. 39, no. 05, pp. 493-496, 2019.

[7] Y. Gu and X. Yang, “Effects of Xueshuantong Injection on platelet parameters, nerve function related factors and nerve function score in patients with posterior circulation cerebral infarction,” *Chinese Journal Of integrative Medicine On Cardio-/Cerebrovascuiar Disease*, vol. 15, no. 19, pp. 2460-2462, 2017.

[8] S. Duan and H. Shang, “Curative effect of Xueshuantong Injection on acute cerebral infarction,” *Practical Journal of Cardiac Cerebral Pneumal and Vascular Disease*, vol. 20, no. 11, pp. 1881, 2012.

[9] J. Ma, “Clinical analysis of 60 cases of acute cerebral infarction treated with Xueshuantong Injection,” *Chinese Medicine Guides*, vol. 7, no. 20, pp. 166-167, 2010.
