# Supplementary material for: Systematic Review and Meta-Analysis on Randomized Controlled Trials on Efficacy and Safety of Panax Notoginseng Saponins in Treatment of Acute Ischemic Stroke
Source: Evid Based Complement Alternat Med. 2021 Jul 9;2021:4694076. doi: 10.1155/2021/4694076 (PMC8289597; doi:10.1155/2021/4694076)
Supplement: Supplementary Materials — Supplementary File 1. Table S1 containing search strategy. Supplementary File 2. Table S2 containing the list of excluded reports. Supplementary File 3. Table S3 containing the basic characteristics of included studies. Supplementary File 4. Table S4 containing the basic characteristics of PNS preparations. Supplementary File 5. Table S5 containing a GRADE summary of outcomes. Supplementary File 6. PRISMA 2020 checklist. Supplementary File 7. Research protocol. [file 4694076.f1.zip › 4694076.f1/Supplementary files 3.docx]

Supplementary Tabel S3: Basic characteristics of included studies

| Study | Sample size | | Male/ female | | Age/ (year) | | Treatment group | | Control group | Duration/ day | Outcomes |
| --- | --- | --- | --- | --- | --- | --- | --- | --- | --- | --- | --- |
|  | T | C | T | C | T | C | PNS | Combined with treatment |  |  |  |
| Zhang Li 2020 [19] | 50 | 50 | 26/24 | 28/22 | 60.46±10.74 | 60.23±10.48 | xueshuantong injection 500 mg/d | CTs | CTs | 14 | ③ |
| Xiao Shuhong 2020 [20] | 39 | 39 | 21/18 | 23/16 | 58.72±7.19 | 58.06±8.27 | xuesaitong injection 400 mg/d | CTs | CTs | 14 | ①④ |
| Wang Juan 2020 [21] | 53 | 53 | 29/24 | 31/22 | 56.13±6.29 | 56.27±6.24 | xuesaitong injection 200 mg/d | CTs | CTs | 14 | ①②⑤ |
| Huang Debo 2020 [22] | 57 | 57 | 31/26 | 33/24 | 63.57±2.18 | 63.61±2.25 | xuesaitong injection 250 mg/d | CTs | CTs | 42 | ①② |
| Zhang Xin 2019 [23] | 49 | 49 | 27/22 | 28/21 | 58.27±5.21 | 58.32±5.27 | xueshuantong injection 450 mg/d | CTs | CTs | 10 | ①②③ |
| Xu Zhimin 2019 [24] | 35 | 35 | 25/10 | 24/11 | 69.1±7.7 | 68.7±7.1 | xueshuantong injection 300 mg/d | CTs | CTs | 14 | ①④ |
| Xiao Jun 2019 [25] | 65 | 65 | 42/23 | 40/25 | 55.16±10.92 | 55.37±10.49 | xuesaitong injection 2 ml/d | CTs | CTs | 14 | ④ |
| Wang Yihong 2019 [26] | 52 | 52 | 25/27 | 27/25 | 55.79±4.52 | 56.42±4.95 | xuesaitong injection 300 mg/d | CTs | CTs | 14 | ① |
| Sun Qiaoshu 2019 [27] | 168 | 168 | 96/72 | 90/78 | 64.2±8.7 | 65.4±8.4 | xuesaitong injection 400 mg/d | CTs | CTs | 14 | ④⑦ |
| Chen Jie 2018 [28] | 25 | 25 | 16/9 | 18/7 | 56.57±13.38 | 57.06±14.87 | xueshuantong injection 2 ml/d | CTs | CTs | 14 | ①④⑧ |
| Tan Wenlan 2018 [29] | 70 | 70 | 42/28 | 39/31 | 62.8±5.2 | 63.41±4.8 | xueshuantong injection 500 mg/d | CTs | CTs | 14 | ①②③⑤ |
| Ren Shan 2018 [30] | 40 | 40 | 31/9 | 33/7 | 64.77±6.98 | 65.95±7.53 | xueshuantong injection 450 mg/d | CTs | CTs | 14 | ② |
| Ouyang juan 2017 [31] | 56 | 56 | 33/23 | 31/25 | 53.6±8.7 | 52.9±8.2 | xuesaitong injection 400 mg/d | CTs | CTs | 14 | ①②⑤ |
| Wang Sujie 2017 [32] | 28 | 28 | 17/11 | 16/12 | 53.91±11.70 | 54.10±11.90 | xuesaitong injection 150 mg/d | CTs | CTs | 14 | ①②⑤ |
| Feng Zhe 2017 [33] | 67 | 67 | 37/30 | 39/27 | 61.95±6.27 | 62.54±5.96 | xueshuantong injection 500 mg/d | CTs | CTs | 21 | ①② |
| Hu Yaozhong 2017 [34] | 56 | 52 | 30/26 | 27/25 | 68.28±7.26 | 67.51±8.33 | xuesaitong injection 400 mg/d | CTs | CTs | 30 | ①②④⑤ |
| Su Yuan 2016 [35] | 40 | 40 | 27/13 | 24/16 | 56.9±5.2 | 57.1±5.7 | xueshuantong injection 400 mg/d | CTs | CTs | 14 | ①②⑤ |
| Zhang Rong 2016 [36] | 48 | 48 | 26/22 | 27/21 | 67.38±3.17 | 68.52±3.64 | xueshuantong injection 300 mg/d | CTs | CTs | 14 | ①⑥ |
| Huang Yuan 2016 [37] | 62 | 62 | 41/21 | 41/21 | 59.2±11.1 | 58.7±11.7 | xuesaitong injection 400 mg/d | CTs | CTs | 14 | ①④ |
| Jiao Yan 2016 [38] | 68 | 67 | 40/28 | 39/28 | 63.24±7.6 | 62.4±7.8 | xueshuantong injection 4 ml/d | CTs | CTs | 14 | ①②③ |
| Zeng Qingli 2015 [39] | 50 | 50 | 29/21 | 27/23 | 61.45±10.47 | 62.37±11.25 | xueshuantong injection 350 mg/d | CTs | CTs | 15 | ①②④⑦⑩ |
| Sun Dan 2015 [40] | 100 | 100 | 54/46 | 57/43 | 62.12±11.39 | 60.34±10.27 | xueshuantong injection 300 mg/d | CTs | CTs | 14 | ①② |
| Han Yan 2014 [41] | 41 | 30 | 26/15 | 22/18 | 72.53±9.24 | 71.45±8.67 | xuesaitong injection 500 mg/d | CTs | CTs | 14 | ①② |
| Tang Duyong 2014 [42] | 21 | 21 | 25/17 | | 65.8±4.7 | | xuesaitong injection 450 mg/d | CTs | CTs | 14 | ① |
| Wang Jiawen 2014 [43] | 30 | 30 | 17/13 | 20/10 | 65.95±10.24 | 66.83±11.34 | xuesaitong injection 400 mg/d | CTs | CTs | 14 | ①②⑤ |
| Yu Yongcai 2014 [44] | 34 | 34 | 18/16 | 20/14 | 58.5±9.7 | 55.1±11.2 | xueshuantong injection 500 mg/d | CTs | CTs | 14 | ①④⑤ |
| Li Naixia 2014 [45] | 34 | 34 | 20/14 | 19/15 | 59.2±9.1 | 58.4±7.6 | xueshuantong injection 500 mg/d | CTs | CTs | 14 | ①③ |
| Cheng Mingxia 2014 [46] | 33 | 30 | 18/15 | 17/13 | 59.8±6.7 | 60.6±6.4 | xuesaitong injection 400 mg/d | CTs | CTs | 14 | ①②⑦⑧ |
| Lu Xiaoping 2014 [47] | 64 | 64 | 55/9 | 52/12 | 57.3±1.4 | 56.8±1.8 | xuesaitong injection 200 mg/d | CTs | CTs | 12 | ⑨ |
| Yang Chengzhi 2013 [48] | 30 | 30 | 32/28 | | 64.5±3.6 | | xuesaitong injection 400 mg/d | CTs | CTs | 10 | ④⑦⑨⑩ |
| Wang Liying 2013 [49] | 65 | 45 | 62/48 | | 40-78 | | xueshuantong injection 500 mg/d | CTs | CTs | 14 | ①④ |
| Zhao Guangfeng 2012 [50] | 56 | 40 | 30/26 | 25/15 | 61.6±14.3 | 61.4±14.4 | xuesaitong injection 800 mg/d | CTs | CTs | 14 | ①② |
| Jiang Ke 2011 [51] | 35 | 35 | - | | 40-75 | | xueshuantong injection 450 mg/d | CTs | CTs | 14 | ①② |
| Luo Xiangdong 2011 [52] | 30 | 30 | 17/13 | 18/12 | 61.37±2.24 | 62.35±8.76 | xuesaitong injection 400 mg/d | CTs | CTs | 14 | ①②⑤ |
| Lei Xiaojuan 2020 [53] | 41 | 41 | 23/18 | 26/15 | 57.90±5.36 | 58.77±6.05 | SQTS 0.6 g/d | CTs | CTs | 15 | ①② |
| Tian Yongqing 2019 [54] | 43 | 42 | 25/18 | 24/18 | 61.55±5.30 | 61.43±5.24 | SQTS 0.6 g/d | CTs | CTs | 28 | ①②⑤ |
| Wei Dongsheng 2017 [55] | 30 | 30 | 18/12 | 19/11 | 58.3±5.6 | 58.7±5.2 | SQTS 0.6 g/d | CTs | CTs | 28 | ①④⑤ |
| Gou Jiyu 2016 [56]  [56] | 48 | 48 | 25/23 | 26/22 | 66.45±7.38 | 66.47±7.39 | SQTS 0.6 g/d | CTs | CTs | 28 | ①②③④⑤ |
| Yang Hongyan 2014 [57] | 40 | 36 | 20/20 | 18/18 | 80.0±7.0 | 81±6.0 | SQTS 0.6 g/d | CTs | CTs | 14 | ①②③④⑤ |
| Li Wei 2018 [58] | 40 | 40 | 22/18 | 20/20 | 60.67±6.79 | 61.37±7.18 | xuesaitong soft capsule 0.24 g/d | CTs | CTs | 56 | ①②⑤ |
| Li Chang 2013 [59] | 36 | 36 | 19/17 | 20/16 | 62 | 60.5 | xuesaitong drop pills 0.15 g/d | CTs | CTs | 15 | ① |
| Wang Qiuru 2017 [60] | 42 | 44 | 25/17 | 27/17 | 67.4±6.6 | 65.6±6.3 | xueshuantong capsule 1.08 g/d | CTs | CTs | 30 | ①② |
| Sun Haijiao 2021 [61] | 43 | 43 | 24/19 | 25/18 | 61.45±7.42 | 60.93±7.11 | xueshuantong injection 450 mg/d | CTs | CTs | 14 | ①②⑤ |

*Note.*①ORR②NDS③ADL-BI④Hemorheology⑤Adverse Reactions⑥3-month Functional Independence Rate

⑦Platelet Parameters⑧MPAR⑨CD62P⑩Coagulation Function
