# Supplementary material for: Systematic Review and Meta-Analysis on Randomized Controlled Trials on Efficacy and Safety of Panax Notoginseng Saponins in Treatment of Acute Ischemic Stroke
Source: Evid Based Complement Alternat Med. 2021 Jul 9;2021:4694076. doi: 10.1155/2021/4694076 (PMC8289597; doi:10.1155/2021/4694076)
Supplement: Supplementary Materials — Supplementary File 1. Table S1 containing search strategy. Supplementary File 2. Table S2 containing the list of excluded reports. Supplementary File 3. Table S3 containing the basic characteristics of included studies. Supplementary File 4. Table S4 containing the basic characteristics of PNS preparations. Supplementary File 5. Table S5 containing a GRADE summary of outcomes. Supplementary File 6. PRISMA 2020 checklist. Supplementary File 7. Research protocol. [file 4694076.f1.zip › 4694076.f1/Supplementary files 4.docx]

Supplementary Tabel S4: Basic characteristics of PNS preparations

| Drug name | Active substance | Price/CNY | Indications | Contraindications |
| --- | --- | --- | --- | --- |
| Xuesaitong Injection^A^ | PNS | 13.00-25.00 | Apoplectic hemiplegia, Arteriosclerotic Thrombotic Cerebral infarction, Cerebral embolism, Central retinal vein occlusion | 1. Ginseng and Panax notoginseng allergy prohibited. 2. It is not allowed to be allergic to this product. 3. It is forbidden in acute stage of hemorrhagic disease. 4. It is forbidden for children. |
| Xuesaitong Injection^B^ | PNS | 9.00-250.00 | Apoplectic hemiplegia, Sequelae of cerebrovascular disease, Chest pain, Central retinal vein occlusion | 1. Acute stage of cerebral hemorrhage. 2. Patients with previous allergy to ginseng and Panax notoginseng. |
| Xueshuantong Injection^A^ | PNS | 1.78-11.18 | Central retinal vein occlusion, Sequelae of cerebrovascular disease, Endophthalmos, Anterior chamber hemorrhage | 1. Ginseng and Panax notoginseng allergy prohibited. 2. It is not allowed to be allergic to this product. 3. It is forbidden in acute stage of hemorrhagic disease. 4. It is forbidden for children. |
| Xueshuantong Injection^B^ | PNS | 31.00-250.00 | Apoplectic hemiplegia, Sequelae of cerebrovascular disease, Chest pain, Central retinal vein occlusion | 1. Ginseng and Panax notoginseng allergy prohibited. 2. It is not allowed to be allergic to this product. 3. It is forbidden in acute stage of hemorrhagic disease. 4. It is forbidden for children. |
| Sanqi Tongshu Capsule | PNS | 15.00-31.68 | Cardio cerebrovascular embolism | 1. Pregnant women. 2. Puerpera. 3. Cerebral hemorrhage. |
| Xuesaitong Soft Capsule | PNS | 17.46-54.24 | Convalescent stage of stroke, Angina pectoris | 1. Pregnant women. |
| Xueshuantong Capsule | PNS | 19.97 | Apoplectic hemiplegia, Chest pain | It's not clear. |
| Xuesaitong Droping Pill | PNS | 24.12-70.77 | Apoplectic hemiplegia, Sequelae of cerebrovascular disease, Chest pain | 1. Pregnant women. |

*Note.*A.liquid;B.Lyophilized Powder Injection.
