# Supplementary material for: Systematic Review and Meta-Analysis on Randomized Controlled Trials on Efficacy and Safety of Panax Notoginseng Saponins in Treatment of Acute Ischemic Stroke
Source: Evid Based Complement Alternat Med. 2021 Jul 9;2021:4694076. doi: 10.1155/2021/4694076 (PMC8289597; doi:10.1155/2021/4694076)
Supplement: Supplementary Materials — Supplementary File 1. Table S1 containing search strategy. Supplementary File 2. Table S2 containing the list of excluded reports. Supplementary File 3. Table S3 containing the basic characteristics of included studies. Supplementary File 4. Table S4 containing the basic characteristics of PNS preparations. Supplementary File 5. Table S5 containing a GRADE summary of outcomes. Supplementary File 6. PRISMA 2020 checklist. Supplementary File 7. Research protocol. [file 4694076.f1.zip › 4694076.f1/Supplementary files 5.docx]

Supplementary Tabel S5: GRADE Summary of Outcomes for PNS+CTs versus CTs for Patients with AIS

| Outments | No. of  Participants (studies) | Anticipated Absolute Effects (95% *CI*) | | Relative Effect (95% *CI*) | Certainty of  the Evidence  (GRADE) |
| --- | --- | --- | --- | --- | --- |
|  |  | Risk with CTs | Risk difference with PNS+CTs |  |  |
| 3-month Functional Independence Rate | 96 (1) | 479 per 1,000 | 417 more per 1,000 (177 more to 743 more) | *RR* 1.87 (1.37 to 2.55) | ⨁◯◯◯ VERY LOW^a,e,d^ |
| Incidence of Adverse Reactions | 629 (8) | 133 per 1,000 | 51 fewer per 1,000 (82 fewer to 4 more) | *RR* 0.62 (0.39 to 0.97) | ⨁⨁◯◯ LOW^a,d^ |
| NIHSS (14d) | 1166 (12) | The mean NIHSS (14d) ranged  from 2.78 to 20.53 | The mean NIHSS (14d) in the PNS+CTs group was 3.94 lower (5.65 lower to 2.23 lower) | - | ⨁◯◯◯ VERY LOW^a,b,d^ |
| ADL-Barthel (14d) | 511 (5) | The mean ADL-Barthel (14d) ranged  from 50.53 to 73.52 | The mean ADL-Barthel (14d) in the PNS+CTs group was 13.92 higher (11.46 higher to 16.38 higher) | - | ⨁⨁◯◯ LOW^a,b,d^ |
| ORR (NIHSS) | 2248 (23) | 773 per 1,000 | 155 more per 1,000 (124 more to 186 more) | *RR* 1.20 (1.16 to 1.24) | ⨁⨁◯◯ LOW^a,d^ |
| WBHSV | 1070 (11) | The mean WBHSV ranged  from 4.78 to 6.86 | The mean WBHSV in the PNS+CTs group was 0.83 lower (1.16 lower to 0.5 lower) | - | ⨁◯◯◯ VERY LOW^a,b,d^ |
| WBLSV | 1024 (10) | The mean WBLSV ranged  from 8.86 to 14.62 | The mean WBLSV in the PNS+CTs group was 1.58 lower (2.41 lower to 0.75 lower) | - | ⨁◯◯◯ VERY LOW^a,b,d^ |
| PV | 1004 (10) | The mean PV ranged  from 1.20 to 2.58 | The mean PV in the PNS+CTs group was 0.4 lower (0.55 lower to 0.25 lower) | - | ⨁◯◯◯ VERY LOW^a,b,d^ |
| FIB | 660 (8) | The mean FIB ranged  from 3.27 to 4.86 | The mean FIB in the PNS+CTs group was 0.84 lower (1.19 lower to 0.49 lower) | - | ⨁◯◯◯ VERY LOW^a,b,d^ |
| MPAR | 113 (2) | The mean MPAR ranged  from 6.37 to 10.41 | The mean MPAR in the PNS+CTs group was 6.82 lower (9.62 lower to 4.02 lower) | - | ⨁◯◯◯ VERY LOW^a,c,d^ |
| PLT | 309 (3) | The mean PLT ranged  from 174.68 to 189 | The mean PLT in the PNS+CTs group was 4.85 higher (1.82 higher to 7.87 higher) | - | ⨁◯◯◯ VERY LOW^a,c,d^ |
| MPV | 249 (2) | The mean MPV ranged  from 8.67 to 12.3 | The mean MPV in the PNS+CTs group was 0.79 lower (1.09 lower to 0.48 lower) | - | ⨁◯◯◯ VERY LOW^a,c,d^ |

*Note.* PNS, Panax Notoginseng Saponins; CTs, conventional treatment; *CI*, Confidence interval; *RR*, relative risks; a, Poor description of methodology including method of randomisation, blinding and outcome assessments; b, *I^2^*≥ 50% for heterogeneity; c, Small number of RCTs with small sample sizes; d, Quantitative evaluation of the included data or small sample indicated that there was the possibility of publication bias; e, Only one study provided data.
