# Supplementary material for: Systematic Review and Meta-Analysis on Randomized Controlled Trials on Efficacy and Safety of Panax Notoginseng Saponins in Treatment of Acute Ischemic Stroke
Source: Evid Based Complement Alternat Med. 2021 Jul 9;2021:4694076. doi: 10.1155/2021/4694076 (PMC8289597; doi:10.1155/2021/4694076)
Supplement: Supplementary Materials — Supplementary File 1. Table S1 containing search strategy. Supplementary File 2. Table S2 containing the list of excluded reports. Supplementary File 3. Table S3 containing the basic characteristics of included studies. Supplementary File 4. Table S4 containing the basic characteristics of PNS preparations. Supplementary File 5. Table S5 containing a GRADE summary of outcomes. Supplementary File 6. PRISMA 2020 checklist. Supplementary File 7. Research protocol. [file 4694076.f1.zip › 4694076.f1/Supplementary files 6.docx]

| **Section and Topic** | **Item #** | **Checklist item** | **Location where item is reported** |
| --- | --- | --- | --- |
| **TITLE** | | |  |
| Title | 1 | Systematic review and Meta-analysis on randomized controlled trials on efficacy and safety of Panax Notoginseng Saponins in treatment of acute ischemic stroke. | Page 1 |
| **ABSTRACT** | | |  |
| Abstract | 2 | Background: Panax Notoginseng Saponins (PNS) proven to have antiplatelet effect in patients with acute ischemic stroke (AIS). We aimed to assess the efficacy and safety of PNS on antiplatelet therapy in the treatment of AIS.  Methods: For this systematic review and meta-analysis, we searched 7 literature databases and 2 clinical studies databases for randomized controlled studies (RCTs) evaluating PNS as an adjuvant therapy for AIS between database inception and December, 2020. Meta-analysis was carried out with the Rev Man 5.4 software. The quality of the included studies was assessed with the Cochrane risk of bias tool. This study is registered on PROSPERO, number CRD42021229265.  Findings: Of 8245 records identified, 42 RCTs met our inclusion criteria (n = 4084 patients) . Patients assigned to PNS with conventional treatments(CTs) had improved functional independence at 90 days compared with those assigned to CTs alone (RR = 1.87, 95% CI = 1.37, to 2.55, P < 0.0001) . Patients who received PNS combined with CTs showed significantly high improvements in neurological function among individuals with AIS on the neurologic deficit score (NDS) (MD_CSS_ = −5.71, 95% CI = −9.55 to −1.87, P = 0.004; MD_NIHSS_ = −3.68, 95% CI = −5.47 to −1.89, P < 0.0001) . The results also showed PNS contributed to a betterment in activities of daily living (ADL) on the Barthel index (MD_day 10 BI_ = 4.86, 95% CI = 2.18, to 7.54, P < 0.00001; MD_day 14 BI_ = 13.92, 95% CI = 11.46 to 16.38, P < 0.00001; MD_day 28 BI_ = 7.16, 95% CI = 0.60, to 13.72, P < 0.00001) . In addition, PNS, compared with CTs alone, could significantly improve overall response rate (ORR) (RR_NIHSS_ = 1.20, 95% CI = 1.16, to 1.24, P < 0.00001; RR_CSS_ = 1.15, 95% CI = 1.08, to 1.24, P < 0.0001), hemorheological parameters, maximum platelet aggregation rate (MPAR) (MD = −6.82, 95% CI = −9.62 to −4.02, P < 0.00001) , platelet parameters (MD_PLT_ =4.85, 95% CI = 1.82 to 7.84, P = 0.002; MDMPV = −0.79, 95% CI = −1.09 to −0.48, P < 0.00001) and serum CD62P (MD = −0.21, 95% CI = −0.29 to −0.13, P < 0.00001) . There was no statistical difference in the incidence of adverse reactions between the PNS and control group (RR = 0.71, 95% CI = 0.44−1.15, P = 0.16) . Adverse reactions in the PNS were mild adverse reactions.  Interpretation: PNS may be effective and safe in treating AIS on ameliorating neurological deficit, improving activities of daily living function and enhancing antiplatelet. However, more high-quality evidence are needed before it can be recommended for routine antiplatelet therapy in patients with AIS.  Funding: General Project of National Natural Science Foundation of China (No.81774159) and 2019 the State Administration of traditional Chinese medicine TCM evidence based capacity building project (ZZ13-024-3). | Page 1 |
| **INTRODUCTION** | | |  |
| Rationale | 3 | Acute ischemic stroke (AIS) , also known as acute cerebral infarction (ICD10 Code: i63. 902) , is a life-threatening medical condition with high incidence that carry grave prognosis if not addressed promptly. At present, conventional treatments recommended by clinical practice guidelines include thrombolytic drugs, antiplatelet drugs, anticoagulants, and neurotrophic drugs. Therefore, reducing the rate of intracranial hemorrhage after reperfusion and overcoming clopidogrel resistance are the requirements of new antiplatelet drugs in the treatment of AIS. The synergistic mechanism of Chinese herbal medicine and antiplatelet drugs of Western medicine has caught worldwide attention. It was found that PNS could enhance the antiplatelet effect by regulating arachidonic acid (AA) metabolic pathway, inhibiting thromboxane A2 (TXA2) or aspirin hydrolase, increasing the AUC_0-∞_ or Cmax of the clopidogrel active metabolite. | Page 2 |
| Objectives | 4 | To evaluate the efficacy and safety of PNS on antiplatelet therapy in the treatment of AIS, to provide the up-to-date evidence for clinical application of PNS. | Page 3 |
| **METHODS** | | |  |
| Eligibility criteria | 5 | Population: We included randomized controlled trials of patients diagnosed with AIS.  Intervention: The experimental group treated with PNS combined with conventional treatments (CTs) and the control group treated with the same CTs. CTs are considered including thrombolytic drugs, antiplatelet, anticoagulant, statins, neuroprotective agents, antihypertensive and collateral circulation drugs.  Efficacy outcomes: The primary outcome was 3-month functional independence rate (mRS scores 0-2), and the [secondary outcomes](http://www.baidu.com/link?url=zuXWsdL8-1J7eKdyEtav6QlIKQ7TGxSO29FccIUaNK0iF-kTL4ISJ_Yypi3fr0Aj2Yw1TJvR6R9I4XeNK5tQnJBNtWSN4SozU8JLDl6oTbICn5qESvHTjSM1pOOoUNnd1Zwk55ryjVQlYXsC82FxBJqWIirRBBsEqpJM3gtIp_Hi6doPPn63kqqz4DSxQPYmCtLIl_Lz36h3FrhAXqgFZ1hQKOMjTRX2B9JixJ71Qg_6Nsj3IVWtvwYV9Ssb_IZPr2UMCaJYYLoCqRKhfVaFHKPNx69IC5S3o5vwG_pWWngghJaMRjCJn-X5NyRHQdyojD0EKOB0TiBx7_DhAKiun_) were neurologic deficit score (NDS) , ADL-Barthel score, overall response rate (ORR) , hemorheological parameters, maximum platelet aggregation rate (MPAR) , platelet parameters, CD62P, and coagulation function.  Safety outcomes: adverse reactions. | Page 4 |
| Information sources | 6 | Database such as CNKI, Wanfang, VIP, CBM, EMBASE, PubMed, Cochrane Library, ClinicalTrials.gov, ChiCTR were searched by research team. A separated database of AIS treated with traditional Chinese medicine was established. The retrieval time is from the establishment of the database to December 2020. Considering that the above search did not explicitly mention PNS, we conducted an additional search by using key words including PNS, Xuesaitong, Xueshuantong, Sanqi Tongshu capsule and Lulutong, and supplement the database of PNS in the treatment of AIS. Taking PubMed as an example, the specific supplementary retrieval strategies are presented in Supplementary Tabel S1. On 4 April 2021, We updated the database search of Pubmed and CNKI, as detailed in the Additional file. We used the same search method, except that we narrowed the searches to 2020 onwards. | Page 3 |
| Search strategy | 7 | The specific supplementary retrieval strategies of all databases are presented in Supplementary Tabel S1.  #1 Cerebral infarction[MeSH Terms]  #2 Cerebral infarction[Title/Abstract]  #3 (((((((((((((((((((((((Cerebral Infarctions[Title/Abstract]) OR (Infarctions, Cerebral[Title/Abstract])) OR (Infarction, Cerebral[Title/Abstract])) OR (Cerebral Infarct[Title/Abstract])) OR (Cerebral Infarcts[Title/Abstract])) OR (Infarct, Cerebral[Title/Abstract])) OR (Cerebral Infarction, Left Hemisphere[Title/Abstract])) OR (Left Hemisphere, Infarction, Cerebral[Title/Abstract])) OR (Infarction, Left Hemisphere, Cerebral[Title/Abstract])) OR (Left Hemisphere, Cerebral Infarction[Title/Abstract])) OR (Cerebral, Left Hemisphere, Infarction[Title/Abstract])) OR (Infarction, Cerebral, Left Hemisphere[Title/Abstract])) OR (Subcortical Infarction[Title/Abstract])) OR (Infarction, Subcortical[Title/Abstract])) OR (Infarctions, Subcortical[Title/Abstract])) OR (Subcortical Infarctions[Title/Abstract])) OR (Posterior Choroidal Artery Infarction[Title/Abstract])) OR (Anterior Choroidal Artery Infarction[Title/Abstract])) OR (Cerebral Infarction, Right Hemisphere[Title/Abstract])) OR (Right Hemisphere, Cerebral Infarction[Title/Abstract])) OR (Infarction, Right Hemisphere, Cerebral[Title/Abstract])) OR (Right Hemisphere, Infarction, Cerebral[Title/Abstract])) OR (Cerebral, Right Hemisphere, Infarction[Title/Abstract])) OR (Infarction, Cerebral, Right Hemisphere[Title/Abstract])  #4 #1 OR #2 OR #3  #5 stroke[MeSH Terms]  #6 stroke[Title/Abstract]  #7 ((((((((((((((((((((((((((Strokes[Title/Abstract]) OR (Cerebrovascular Accident[Title/Abstract])) OR (CVA (Cerebrovascular Accident)[Title/Abstract])) OR (CVAs (Cerebrovascular Accident)[Title/Abstract])) OR (Cerebrovascular Apoplexy[Title/Abstract])) OR (Apoplexy, Cerebrovascular[Title/Abstract])) OR (Vascular Accident, Brain[Title/Abstract])) OR (Brain Vascular Accident[Title/Abstract])) OR (Brain Vascular Accidents[Title/Abstract])) OR (Brain Vascular Accidents[Title/Abstract])) OR (Cerebrovascular Stroke[Title/Abstract])) OR (Cerebrovascular Strokes[Title/Abstract])) OR (Stroke, Cerebrovascular[Title/Abstract])) OR (Strokes, Cerebrovascular[Title/Abstract])) OR (Apoplexy[Title/Abstract])) OR (Cerebral Stroke[Title/Abstract])) OR (Cerebral Strokes[Title/Abstract])) OR (Stroke, Cerebral[Title/Abstract])) OR (Strokes, Cerebral[Title/Abstract])) OR (Stroke, Acute[Title/Abstract])) OR (Acute Stroke[Title/Abstract])) OR (Acute Strokes[Title/Abstract])) OR (Strokes, Acute[Title/Abstract])) OR (Cerebrovascular Accident, Acute[Title/Abstract])) OR (Acute Cerebrovascular Accident[Title/Abstract])) OR (Acute Cerebrovascular Accidents[Title/Abstract])) OR (Cerebrovascular Accidents, Acute[Title/Abstract])  #8 #5 OR #6 OR #7  #9 #4 OR #8  #10 (((((((((Panax notoginseng extract[Title/Abstract]) OR (Xueshuantong[Title/Abstract])) OR (Xue shuan tong[Title/Abstract])) OR (Xuesetong[Title/Abstract])) OR (xuesaitong[Title/Abstract])) OR (xuesetong injection[Title/Abstract])) OR (xue sai tong[Title/Abstract])) OR (sanqi[Title/Abstract])) OR (Sanqi Tongshu capsule[Title/Abstract])) OR (Lulutong[Title/Abstract])  #11 #9 AND #10 | Page 3 |
| Selection process | 8 | Two reviewers (LDW and ZMX) independently performed literature selection according to the predefined eligibility criteria. The records retrieved in all databases were imported into NoteExpress3.2, and the duplicated records were deleted. Records were first screened based on the title and abstract, and in cases of uncertainty, the full texts were obtained. Any disagreement between the paired reviewers was resolved through discussing with a third reviewer (XL) . | Page 4 |
| Data collection process | 9 | Data extraction was conducted by two reviewers (LDW and WRQ) using a standardized, predetermined data extraction form. Two reviewers independently extracted data from each trial, and then cross-checked the data. Discrepancies were solved by discussing within the two reviewers or arbitrated by the senior researcher (XL) if necessary. We extracted the following data: 1) study characteristics; 2) participant's baseline characteristics and inclusion/exclusion criteria; 3) details of intervention and control groups; and 4) outcomes (dichotomous data were number of events and total participants per group; continuous data were presented as mean, standard deviation, and total participants per group) . | Page 4-5 |
| Data items | 10a | Eligible outcomes were broadly categorised as follows:  Functional independence  Neurologic deficit score  Laboratory indexes related to antiplatelet  Any measure of functional independence, neurologic deficit score was eligible for inclusion. For laboratory indexes related to antiplatelet, we gave priority to maximum platelet aggregation rate (MPAR) , platelet parameters and coagulation function. | Page 1 |
|  | 10b | We collected data on:  the report: author, year;  the study: sample characteristics;  the research design and features: random sequence generation, allocation concealment, adherence, blinding, and length of follow up;  the intervention: type, duration, dose. | Page 5 |
| Study risk of bias assessment | 11 | Two reviewers (LDW and SJL) independently assessed the risk of bias of the included trials. According to the Cochrane Risk of Bias tool [17] , seven fields of risk of bias were evaluated as below: random sequence generation, allocation concealment, blinding of participants and personnel, blinding of outcome assessment, incomplete outcome data, selective reporting, and other bias. The evaluation results were ranked with low risk, unclear risk or high risk. If disagreements on the assessment were identified, the researcher (XL) was consulted. | Page 5 |
| Effect measures | 12 | Risk ratio (RR) were used for dichotomous data while weighted mean difference (WMD) or standardized mean difference (SMD) were adopted for continuous variables as effect size, both of which were demonstrated with effect size and 95% confidence intervals (CI) . | Page 5 |
| Synthesis methods | 13a | The intervention in all included studies were conventional treatments and Panax notoginseng saponins, and the heterogeneity of demographic information of the participants was small, therefore we decided which studies were eligible for each synthesis only based on the outcomes. | Page 5 |
|  | 13b | The original data extracted from reports were used instead of data transformation. | Page 5 |
|  | 13c | We used forest plot to visually display results of meta syntheses and use study characteristic table to tabulate the baseline information of included studies. | Page 5 |
|  | 13d | We tested heterogeneity in Review Manager 5.4. When no statistical heterogeneity was identified (heterogeneity test, P ≥ 0.10, or I2 ≤ 50%) , fixed-effects model was selected, otherwise random-effects model was applied. | Page 5 |
|  | 13e | We performed subgroup analyses based on the course of treatment or dose, or follow-up time. Sources of heterogeneity were be fully explored. | Page 5 |
|  | 13f | We conducted sensitivity analysis, and we found that the study sites of two studies were quite different from those of three other studies, which were probably the major source of the heterogeneity. Tongliao and Urumqi were the sites of two studies, with dimensions of 43.6 and 43.4 degrees north latitude respectively, far north of the other three cities Xi'an, Shanghai and Nanning. We considered that this was related to the influence of regional climate on blood viscosity, which will be further explored in the future. | Page 5 |
| Reporting bias assessment | 14 | To assess small-study effects, we planned to generate funnel plots for meta-analyses including at least 10 trials of varying size. To assess outcome reporting bias, we compared the outcomes specified in trial protocols with the outcomes reported in the corresponding trial publications; if trial protocols were unavailable, we compared the outcomes reported in the methods and results sections of the trial publications. | Page 5 |
| Certainty assessment | 15 | Two reviewers (LDW and CYG) independently assessed the certainty of the evidence using the Grading of Recommendations Assessment, Development and Evaluation (GRADE) approach , and assessed the certainty of the evidence as high, moderate, low, or very low. The certainty can be downgraded for five GRADE considerations (study limitations, consistency of effect, imprecision, indirectness, and publication bias) and upgraded for three reasons (large magnitude of an effect, dose-response gradient, and effect of plausible residual confounding) . | Page 6 |
| **RESULTS** | | |  |
| Study selection | 16a | The search yielded 8245 records. There were 5015 duplicates, leaving 3230 to be screened by title and abstract from which 80 eligible studies were retained for full-text evaluation. After careful evaluation and no disagreements between the two reviewers, 38 studies were excluded. Ultimately, 42 trials involving 4084 participants met our inclusion criteria . | Page 6 |
|  | 16b | A list of studies that might appear to meet the inclusion criteria but which were excluded, with citation and the reason for exclusion, are reported in Supplementary Tabel S2. | Page 6 |
| Study characteristics | 17 | We included a table presenting for each included study the sample size, mean age, sex ratio, Interventions, durations and outcomes (table 1) . | Page 8-11 |
| Risk of bias in studies | 18 | We have summarized risks of bias in the included trials in Figure 2. For “random sequence generation” , we rated twenty-four trials as having low risk of selection bias because the authors reported suitable randomization process, of which twenty-one trials ] used random number table, two trials used systematic random, and one trail used lottery. Thirteen trials had unclear risk of bias for this domain due to the lack of an adequate description of how the random sequence generation was conducted. Three trials had high risk of bias for the domain due to the random sequence generated by admission date and admission order . For “allocation concealment” , we considered risk of bias to be unclear in forty-two trials , on account of the lack of reporting the allocation concealment methodology. For “blinding” , we rated one trial as having low risk of bias because the authors explicitly reported blinding was implemented. And we judged forty-one trials as unclear risk due to the absence of information regarding blinding of participants, personnel and outcome assessment. For “incomplete outcome data” , we rated risk of attrition bias as unclear in forty-two trials, because the authors did not mention the loss of follow-up. For “selective reporting” , we considered forty-one trials as having a low risk of bias due to reported the preset outcomes. We judged one trials as having a high risk of reporting bias because the appropriate data about the pre-designed outcome were unavailable. It is not clear whether there are other biases. | Page 13-14 |
| Results of individual studies | 19 | For an example of individual study results presented for a dichotomous outcome, see [figure 2](https://www.bmj.com/content/372/bmj.n160#F2). For an example of individual study results presented for a continuous outcome, see [figure 3](https://www.bmj.com/content/372/bmj.n160#F3). 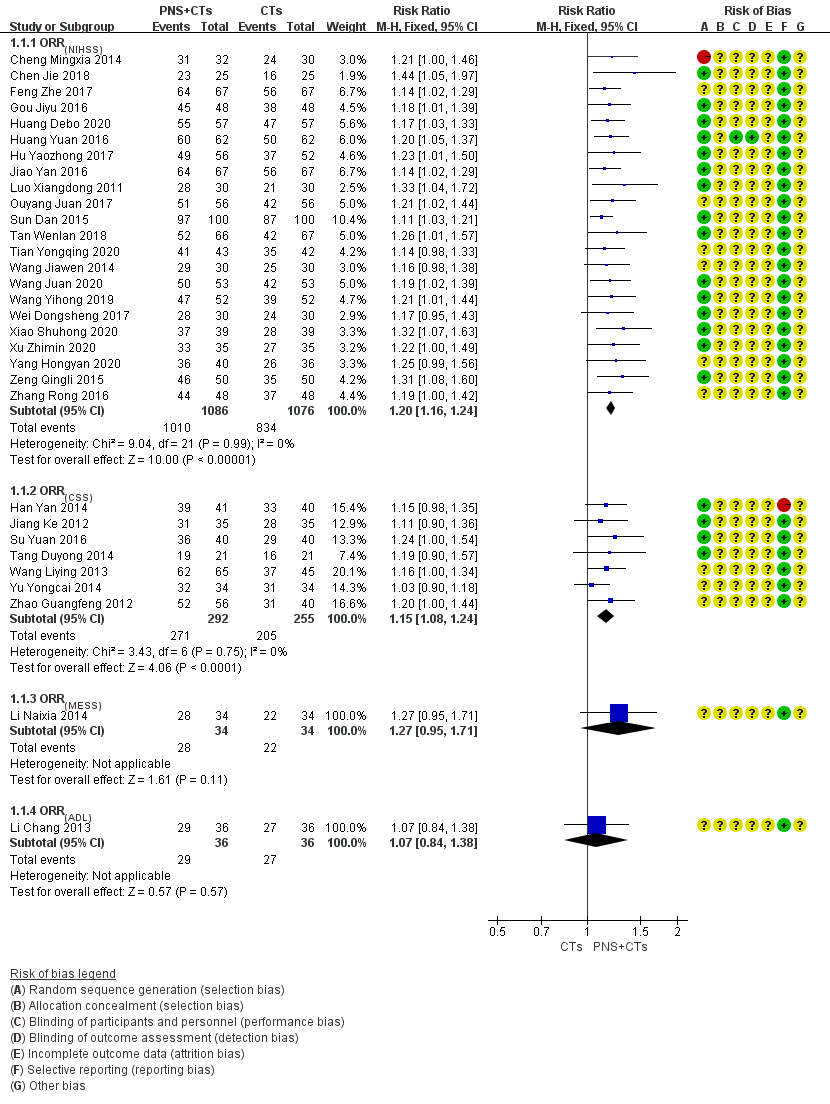 Fig 8 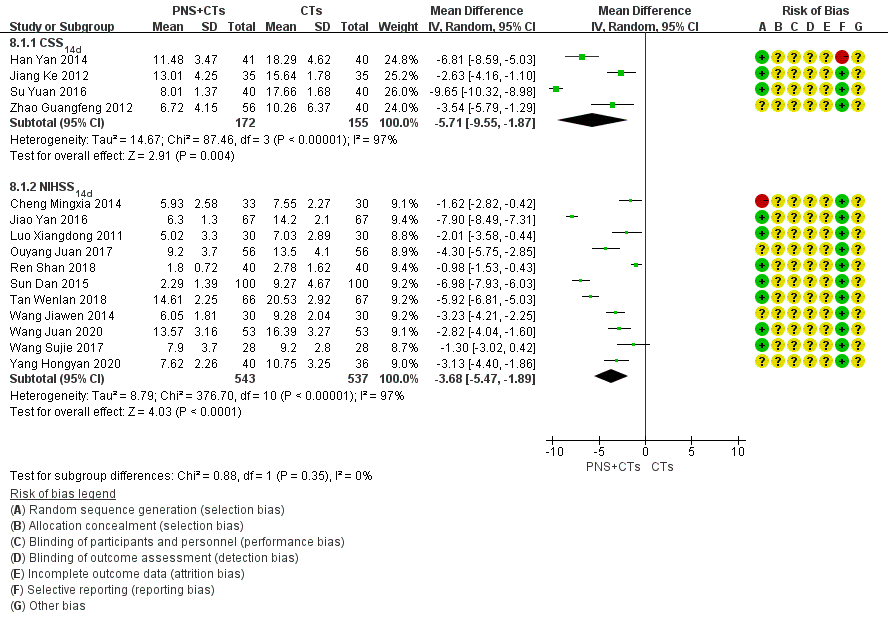 Fig 5 | Page 14-26 |
| Results of syntheses | 20a | A total of seven studies measured the changes in the ADL-Barthel score. These RCTs enrolled 705 patients. Four of the trials had low risk of selection bias, three had unclear risk of selection bias. For “selective reporting” , seven of the trials had low risk of bias. | Page 14-26 |
|  | 20b | Seven studies, including a total of 705 patients, reported ADL-Barthel score. PNS plus CTs, as compared with CTs independently, was associated with a significant improvement in ADL-Barthel score (MDday 10 BI = 4.86, 95% CI = 2.18, to 7.54, P < 0.00001; MDday 14 BI = 13.92, 95% CI = 11.46 to 16.38, P < 0.00001; MDday 28 BI = 7.16, 95% CI = 0.60, to 13.72, P < 0.00001) . | Page 14-26 |
|  | 20c | The significant heterogeneity was identified among the studies (I^2^ = 50%, P = 0.09) , a random-effects model was useds. We found that the study sites of two studies [19, 38] were quite different from those of three other studies, which were probably the major source of the heterogeneity. | Page 14-26 |
|  | 20d | Sensitivity analyses that removed studies with potential bias showed consistent results with the primary meta-analyses. | Page 14-26 |
| Reporting biases | 21 | The ORR_NIHSS_ of twenty-two studies was evaluated by the funnel chart, and the results showed that the left-right asymmetry may be related to the low methodological quality and unpublished negative results of the included studies. | Page 26-27 |
| Certainty of evidence | 22 | The GRADE system was used to assess the level of evidence for the twelve outcomes, which indicated low or very low quality with serious methodological problems, a heterogeneity problem and a small sample problem. | Page 27-29 |
| **DISCUSSION** | | |  |
| Discussion | 23a | Most of the existing systematic reviews observed a certain kind of PNS preparations , and pay more attention to the efficacy of PNS combined with a certain western medicine, such as Xueshuantong combined with edaravone or butylphthalide, but lack of the latest clinical research results to evaluate the therapeutic effect of PNS as the only variable in the intervention and control group.  Compared with the previous reviews, the current systematic reveiw is comprehensive and included 42 trials, which provides relatively complete and up-to -date evidence on the use of PNS as adjunctive therapies for AIS. We used an evidence-based medicine approach to critically review the existing evidence from previous RCTs, and we found a better effect of PNS for independent function, platelet parameters and MPAR. In addition, we applied GRADE criteria to determine the certainty in the estimate of effect for important outcomes. | Page 3 and 31 |
|  | 23b | The quality of the included trials is generally poor in random sequence generation and blind design, which is a common problem in the current situation of clinical trials of TCM. In addition, the insufficient sample size of included studies in some comparisons affected the reliability of the results. | Page 31 |
|  | 23c | Firstly, in the real world various drugs are commonly used in the treatment of AIS. Although we strictly limited the drug category of CTs in the eligibility criteria, in our review, most of trials did not mention the specific therapeutic regimen, which resulted in inevitable clinical heterogeneity to a certain extent. Furthermore, excessive statistical heterogeneity came to our notice in some of the comparisons. However, we cannot identify the source of heterogeneity through the data and information provided. | Page 31 |
|  | 23d | Long-term outcome, such as 3-month favorable functional outcome, should be chosen as the primary outcome, instead of using intermediate outcomes to substitute for endpoint outcomes as many clinical trials of TCM. The measurement time of various outcomes should be standardized to ensure the data merging between different studies. NIHSS score are suggested to be used in evaluating the neurological deficit uniformly, in order to avoid the heterogeneity caused by different standards. It is hoped that more attention will be paid to the occurrence of bleeding events during the treatment of AIS, since the combination of antiplatelet drugs, anticoagulants and PNS makes it difficult to evaluate the bidirectional regulation function only through laboratory indicators such as MPAR, MPV and PT. Future researchers are urged to design experiments based on rigorous methodology, including appropriate sample sizes and adequate follow-up with long-term duration, and the standardized report will be carried out according to the guidelines of SPIRIT-TCM Extension 2018 and CONSORT-CHM Formulas 2017. In terms of safety analysis, researchers must assess whether the adverse events are related to drug use. And economic analysis should be considered to guide practices. | Page 31 |
| **OTHER INFORMATION** | | |  |
| Registration and protocol | 24a | This systematic review has been registered in the international prospective register of systematic reviews (PROSPERO) under the registration number: CRD42021229265. | Page 3 |
|  | 24b | This systematic review and meta-analysis protocol has been provided in additional file. | Page 3 |
|  | 24c | The search date in the protocol is December 2020, but now it is April 2021, so we updated the database search of Pubmed and CNKI. |  |
| Support | 25 | The study was funded by the General Project of National Natural Science Foundation of China (No.81774159) and 2019 the State Administration of traditional Chinese medicine TCM evidence based capacity building project (ZZ13-024-3). Xing Liao provided project oversight, reviewed the report to ensure that the analysis met methodological standards. | Page 32-33 |
| Competing interests | 26 | No conflicts of interests. | Page 32 |
| Availability of data, code and other materials | 27 | The data used in the article are obtained from public databases. The process including the literature, data extraction, and calculation are all described in the article. If necessary, the first author LDW (liudingwang97@163.com) can be contacted to obtain data. | Page 32 |

*From:*  Page MJ, McKenzie JE, Bossuyt PM, Boutron I, Hoffmann TC, Mulrow CD, et al. The PRISMA 2020 statement: an updated guideline for reporting systematic reviews. BMJ 2021;372:n71. doi: 10.1136/bmj.n71

For more information, visit: <http://www.prisma-statement.org/>
