# Supplementary material for: Systematic Review and Meta-Analysis on Randomized Controlled Trials on Efficacy and Safety of Panax Notoginseng Saponins in Treatment of Acute Ischemic Stroke
Source: Evid Based Complement Alternat Med. 2021 Jul 9;2021:4694076. doi: 10.1155/2021/4694076 (PMC8289597; doi:10.1155/2021/4694076)
Supplement: Supplementary Materials — Supplementary File 1. Table S1 containing search strategy. Supplementary File 2. Table S2 containing the list of excluded reports. Supplementary File 3. Table S3 containing the basic characteristics of included studies. Supplementary File 4. Table S4 containing the basic characteristics of PNS preparations. Supplementary File 5. Table S5 containing a GRADE summary of outcomes. Supplementary File 6. PRISMA 2020 checklist. Supplementary File 7. Research protocol. [file 4694076.f1.zip › 4694076.f1/Supplementary files 7.pdf]

National Institute for Health Research

UNIVERSITY of York
Centre for Reviews and Dissemination

Systematic review

1. * Review title.

Give the title of the review in English

Efficacy and Safety of Panax Notoginseng Saponins in Patients with Acute Ischemic Stroke: A Systematic Review and Meta-Analysis of Randomized Clinical Trials

2. Original language title.

For reviews in languages other than English, give the title in the original language. This will be displayed with the English language title.

3. * Anticipated or actual start date.

Give the date the systematic review started or is expected to start.

22/12/2020

4. * Anticipated completion date.

Give the date by which the review is expected to be completed.

02/02/2021

5. * Stage of review at time of this submission.

Tick the boxes to show which review tasks have been started and which have been completed. Update this field each time any amendments are made to a published record.

Reviews that have started data extraction (at the time of initial submission) are not eligible for inclusion in PROSPERO. If there is later evidence that incorrect status and/or completion date has been supplied, the published PROSPERO record will be marked as retracted.

This field uses answers to initial screening questions. It cannot be edited until after registration.

The review has not yet started: No

Review stage	Started	Completed
Preliminary searches	Yes	No
Piloting of the study selection process	Yes	No
Formal screening of search results against eligibility criteria	No	No
Data extraction	No	No
Risk of bias (quality) assessment	No	No
Data analysis	No	No

Provide any other relevant information about the stage of the review here.

6. * Named contact.

The named contact is the guarantor for the accuracy of the information in the register record. This may be any member of the review team.

Wang Liu Ding

Email salutation (e.g. "Dr Smith" or "Joanne") for correspondence:

Dr Liu Ding

7. * Named contact email.

Give the electronic email address of the named contact.

liudingwang97@163.com

8. Named contact address

Give the full institutional/organisational postal address for the named contact.

Xiyuan Hospital of Haidian District, Beijing

9. Named contact phone number.

Give the telephone number for the named contact, including international dialling code.

18562699156

10. * Organisational affiliation of the review.

Full title of the organisational affiliations for this review and website address if available. This field may be completed as 'None' if the review is not affiliated to any organisation.

Xiyuan Hospital

Organisation web address:

http://www.xyhospital.com/

11. * Review team members and their organisational affiliations.

Give the personal details and the organisational affiliations of each member of the review team. Affiliation refers to groups or organisations to which review team members belong. **NOTE: email and country now MUST be entered for each person, unless you are amending a published record.**

Dr Wang Liu ding. Xiyuan Hospital
Professor LIAO Xing. Center for Evidence-based Chinese Medicine, Institute of Basic Research in Clinical Medicine, China Academy of Chinese Medical Sciences, Beijing
Professor ZHANG Yunling. Xiyun Hospital
XU Zhenmin. Xiyuan Hospital

12. * Funding sources/sponsors.

Details of the individuals, organizations, groups, companies or other legal entities who have funded or sponsored the review.

Evidence based capacity building project of traditional Chinese medicine of State Administration of traditional Chinese medicine in 2019

Grant number(s)

State the funder, grant or award number and the date of award

ZZ13-024-3

13. * Conflicts of interest.

List actual or perceived conflicts of interest (financial or academic).

None

14. Collaborators.

Give the name and affiliation of any individuals or organisations who are working on the review but who are not listed as review team members. **NOTE: email and country must be completed for each person, unless you are amending a published record.**

15. * Review question.

State the review question(s) clearly and precisely. It may be appropriate to break very broad questions down into a series of related more specific questions. Questions may be framed or refined using PI(E)COS or similar where relevant.

We aimed to examine the efficacy and safety of Panax Notoginseng Saponins use in patients with acute ischemic stroke.

16. * Searches.

State the sources that will be searched (e.g. Medline). Give the search dates, and any restrictions (e.g. language or publication date). Do NOT enter the full search strategy (it may be provided as a link or attachment below.)

We will search articles in four Chinese databases(CNKI, VIP, Wan Fang, Sino Med), three English databases(PubMed, EMbase, Cochrane Library) and two Clinical trial registries(ChiCTR, Clinical Trail.gov).

All the publications until December 2020 will be searched without any restriction of countries or article type.

17. URL to search strategy.

Upload a file with your search strategy, or an example of a search strategy for a specific database, (including the keywords) in pdf or word format. In doing so you are consenting to the file being made publicly accessible. Or provide a URL or link to the strategy. Do NOT provide links to your search **results**.

Alternatively, upload your search strategy to CRD in pdf format. Please note that by doing so you are consenting to the file being made publicly accessible.

Do not make this file publicly available until the review is complete

18. * Condition or domain being studied.

Give a short description of the disease, condition or healthcare domain being studied in your systematic review.

Acute ischemic stroke (AIS) is a type of stroke with high incidence, high disability rate and high mortality, characterized by acute onset. According to epidemiological studies, there are about 17 million patients with AIS in the world every year , 6.2 million people died of AIS, and the death / disability rate of hospitalized patients with AIS in China is 34.5% ~ 37.1% within 3 months after onset .

19. * Participants/population.

Specify the participants or populations being studied in the review. The preferred format includes details of both inclusion and exclusion criteria.

Inclusion criteria?

(1)Adults with acute ischemic stroke will be included.(2) Examined the use of Panax Notoginseng Saponins in combination with conventional therapy. (3)Randomized controlled study.

Exclusion criteria?

(1)There were other differences between intervention group and control group except Panax notoginseng saponins.(2)It contains other traditional Chinese medicine preparations except Panax notoginseng saponins.

20. * Intervention(s), exposure(s).

Give full and clear descriptions or definitions of the interventions or the exposures to be reviewed. The preferred format includes details of both inclusion and exclusion criteria.

Panax Notoginseng Saponins was the main intervention (e.g. Xuesaitong injection, Xueshuantong injection, Lulutong injection, Xuesaitong capsules, Xueshuantong capsules, Sanqi Tongshu capsules, Xuesaitong dropping pills).

21. * Comparator(s)/control.

Where relevant, give details of the alternatives against which the intervention/exposure will be compared (e.g. another intervention or a non-exposed control group). The preferred format includes details of both inclusion and exclusion criteria.

The intervention measures of the control group were basic treatment of Western medicine, including improving cerebral blood circulation, lowering blood fat and neuroprotection.

22. * Types of study to be included.

Give details of the study designs (e.g. RCT) that are eligible for inclusion in the review. The preferred format includes both inclusion and exclusion criteria. If there are no restrictions on the types of study, this should be stated.

Randomized controlled trials (RCTs) will be included.

23. Context.

Give summary details of the setting or other relevant characteristics, which help define the inclusion or exclusion criteria.

24. * Main outcome(s).

Give the pre-specified main (most important) outcomes of the review, including details of how the outcome is defined and measured and when these measurement are made, if these are part of the review inclusion criteria.

3-month functional independence (FI; mRS scores 0–2)

* Measures of effect

Please specify the effect measure(s) for you main outcome(s) e.g. relative risks, odds ratios, risk difference, and/or 'number needed to treat.

relative risks(RR)

25. * Additional outcome(s).

List the pre-specified additional outcomes of the review, with a similar level of detail to that required for main outcomes. Where there are no additional outcomes please state 'None' or 'Not applicable' as appropriate to the review

Evaluation of neurological deficit, activities of daily living, total effective rate, hemorheological indexes, platelet parameters(PLT, PDW, MPV), maximum platelet aggregation rate (MPAR) , coagulation function and Platelet activation markers(CD62p).

* Measures of effect

Please specify the effect measure(s) for you additional outcome(s) e.g. relative risks, odds ratios, risk difference, and/or 'number needed to treat.

Total effective rate is RR, and other outcomes are MD.

26. * Data extraction (selection and coding).

Describe how studies will be selected for inclusion. State what data will be extracted or obtained. State how this will be done and recorded.

Two authors will independently extract data. Any disagreement will be resolved by discussion until consensus is reached or by consulting a third author.The following data will be extracted: author, year of publication, total number of people included in the study, doses of Panax notoginseng preparation and time of application.

27. * Risk of bias (quality) assessment.

State which characteristics of the studies will be assessed and/or any formal risk of bias/quality assessment tools that will be used.

We subsequently assessed the quality of included studies using the Cochrane Risk of Bias tool, which assesses studies based upon seven domains. These include: random sequence generation, allocation concealment, blinding of participants and personnel, blinding of outcome assessment, incomplete outcome

data, selective reporting, and other sources of bias.

28. * Strategy for data synthesis.

Describe the methods you plan to use to synthesise data. This **must not be generic text** but should be **specific to your review** and describe how the proposed approach will be applied to your data. If meta-analysis is planned, describe the models to be used, methods to explore statistical heterogeneity, and software package to be used.

Revman 5.4 software provided by Cochrane Collaboration Network was used for meta analysis. Statistical heterogeneity was assessed using I^2 statistics.

29. * Analysis of subgroups or subsets.

State any planned investigation of ‘subgroups’. Be clear and specific about which type of study or participant will be included in each group or covariate investigated. State the planned analytic approach.

For the same outcome index, different scales were used for subgroup analysis.Subgroup analysis should also be carried out for the studies with different courses of treatment that have great influence on the results of the trial.

30. * Type and method of review.

Select the type of review, review method and health area from the lists below.

Type of review

Cost effectiveness
No

Diagnostic
No

Epidemiologic
No

Individual patient data (IPD) meta-analysis
No

Intervention
Yes

Meta-analysis
Yes

Methodology
No

Narrative synthesis
No

Network meta-analysis
No

Pre-clinical
No

Prevention
No

Prognostic

No

Prospective meta-analysis (PMA)
No

Review of reviews
No

Service delivery
No

Synthesis of qualitative studies
No

Systematic review
Yes

Other
No

Health area of the review

Alcohol/substance misuse/abuse
No

Blood and immune system
No

Cancer
No

Cardiovascular
No

Care of the elderly
No

Child health
No

Complementary therapies
No

COVID-19
No

Crime and justice
No

Dental
No

Digestive system
No

Ear, nose and throat
No

Education
No

Endocrine and metabolic disorders
No

Eye disorders
No

General interest
No

Genetics
No

Health inequalities/health equity
No

Infections and infestations
No

International development
No

Mental health and behavioural conditions
No

Musculoskeletal
No

Neurological
Yes

Nursing
No

Obstetrics and gynaecology
No

Oral health
No

Palliative care
No

Perioperative care
No

Physiotherapy
No

Pregnancy and childbirth
No

Public health (including social determinants of health)
No

Rehabilitation
No

Respiratory disorders
No

Service delivery
No

Skin disorders
No

Social care

No

Surgery
No

Tropical Medicine
No

Urological
No

Wounds, injuries and accidents
No

Violence and abuse
No

31. Language.

Select each language individually to add it to the list below, use the bin icon to remove any added in error.
English

There is not an English language summary

32. * Country.

Select the country in which the review is being carried out. For multi-national collaborations select all the countries involved.

China

33. Other registration details.

Name any other organisation where the systematic review title or protocol is registered (e.g. Campbell, or The Joanna Briggs Institute) together with any unique identification number assigned by them. If extracted data will be stored and made available through a repository such as the Systematic Review Data Repository (SRDR), details and a link should be included here. If none, leave blank.

34. Reference and/or URL for published protocol.

If the protocol for this review is published provide details (authors, title and journal details, preferably in Vancouver format)

Add web link to the published protocol.

Or, upload your published protocol here in pdf format. Note that the upload will be publicly accessible.

No I do not make this file publicly available until the review is complete

Please note that the information required in the PROSPERO registration form must be completed in full even if access to a protocol is given.

35. Dissemination plans.

Do you intend to publish the review on completion?

Yes

Give brief details of plans for communicating review findings.?

36. Keywords.

Give words or phrases that best describe the review. Separate keywords with a semicolon or new line. Keywords help PROSPERO users find your review (keywords do not appear in the public record but are included in searches). Be as specific and precise as possible. Avoid acronyms and abbreviations unless these are in wide use.

37. Details of any existing review of the same topic by the same authors.

If you are registering an update of an existing review give details of the earlier versions and include a full bibliographic reference, if available.

38. * Current review status.

Update review status when the review is completed and when it is published.New registrations must be ongoing so this field is not editable for initial submission.
Please provide anticipated publication date

Review_Ongoing

39. Any additional information.

Provide any other information relevant to the registration of this review.

40. Details of final report/publication(s) or preprints if available.

Leave empty until publication details are available OR you have a link to a preprint (NOTE: this field is not editable for initial submission). List authors, title and journal details preferably in Vancouver format.

Give the link to the published review or preprint.
